# The Ability to Direct Attention in Working Memory Is Not Impaired in Adults With Symptoms of ADHD

**DOI:** 10.1177/10870547251330039

**Published:** 2025-04-18

**Authors:** Amy L. Atkinson, Beatriz Pinheiro Sanchez, Matthew Warburton, Heather Allmark, Richard J. Allen

**Affiliations:** 1 Lancaster University, UK; 2 University of Leeds, UK

**Keywords:** working memory, attention, reward, value-based prioritization, ADHD

## Abstract

**Objective:**

Neurotypical individuals can prioritize particularly valuable information in working memory. This is a well-replicated effect, demonstrated across a wide variety of task factors and age groups. However, it is not clear if individuals with symptoms of ADHD are able to do this effectively, as there is some evidence this group struggle to allocate attention in working memory tasks. Two experiments were conducted online to investigate this.

**Method:**

Participants were presented with series of four colored shapes, and asked to report the color of each shape in a counterbalanced order following a brief delay. In some trials (equal value condition), all shapes were equally valuable with the correct recall of each shape gaining the participant 2 points. In other trials (differential value condition), the first item presented during the encoding phase was more valuable than the rest (5 point for the first item vs. 1 point for the other items). Trial-by-trial feedback was either provided (Experiment 1) or omitted (Experiment 2).

**Results:**

Across both experiments, there was a clear prioritization effect at the first (targeted) serial position, with higher accuracy in the differential value condition relative to the equal value condition. There were also clear costs at the less valuable serial positions. These effects did not differ as a function of ADHD symptoms. There were also no significant correlations between scores on the Adult ADHD Self-Report Screener and the prioritization effects.

**Conclusion:**

Taken together, these findings demonstrates that the ability to prioritize particularly valuable information in working memory is not impaired in individuals with symptoms of ADHD.

Working memory refers to an individual’s ability to store and process a limited amount of information for a brief period of time (e.g., seconds; Baddeley et al., 2021; Cowan, 2017). It is considered crucial for a range of everyday activities, including following instructions (Gathercole et al., 2006; Waterman et al., 2017), reading comprehension (Cain et al., 2004; Cowan, 2014), and mental arithmetic (Fürst & Hitch, 2000). Working memory abilities are subject to large individual differences (Alloway, 2006), and working memory difficulties often co-occur with a range of neurodevelopmental diagnoses (Ramos et al., 2020; Smith-Spark & Fisk, 2007), including ADHD (Ramos et al., 2020). ADHD is characterized by elevated levels of inattention and/or hyperactivity, and affects over 7% of children worldwide (Thomas et al., 2015), with symptoms often persisting into adulthood (Faraone et al., 2006; Kessler et al., 2006; Sibley et al., 2016, 2017).

It has been suggested that working memory may be a key area of cognitive dysfunction in ADHD (Ramos et al., 2020), and potentially a core deficit of the disorder (Kofler et al., 2008; Rapport et al., 2009). Supporting this, clear difficulties in working memory tasks have been observed, both during development and in adulthood (e.g., Alderson et al., 2013; Kasper et al., 2012; Martinussen et al., 2005; Ramos et al., 2020; Yang et al., 2017). For example, a meta-analysis investigating working memory difficulties in children with ADHD found moderate-large impairments in both spatial and verbal working memory, with difficulties particularly prevalent with storage and processing of spatial information (Martinussen et al., 2005). Furthermore, a meta-analytic review examining working memory difficulties in adults with ADHD found impairments in both phonological and visuo-spatial working memory (Alderson et al., 2013). Taken together, these studies provide clear evidence of difficulties with working memory tasks in individuals with ADHD, which may persist into adulthood (Alderson et al., 2013; Martinussen et al., 2005). There is also evidence that difficulties in working memory in individuals with ADHD may be more associated with key outcomes than the inattentive and hyperactive symptoms themselves (e.g., Simone et al., 2018). For example, Simone et al. (2018) found that working memory difficulties in children with ADHD were more predictive of academic achievement than their symptoms of ADHD.

One key question of interest in neurotypical populations in recent years has been the extent to which individuals can direct their attention to particularly important or goal-relevant information in working memory (see Allen et al., 2024, Souza & Oberauer, 2016, for reviews). This question is of theoretical interest, as it reflects the extent to which individuals can use their limited working memory resources to prioritize particularly relevant information. It is also of practical interest, potentially revealing a novel approach to ensuring that important information is retained. One key approach used to examine this has been to manipulate an item’s value, such that participants gain more notional points for recall of some items relative to others. In this paradigm, participants are typically presented with sets of colored shapes to recall after a brief delay. Prior to encoding, participants are informed how many points an item is worth if they are asked about that item and they respond correctly. In some trials, they may be told that all items are equally valuable (e.g., worth 1 points). In other trials, participants may be told that correct recall of a particular item will gain them more points than the rest of the items (e.g., 4 points for the high value item, vs. 1 point for the low value items). Using this approach, it has been demonstrated that individuals can prioritize particularly valuable information in working memory, as evidenced by enhanced recall for higher value items (e.g., Atkinson et al., 2018; Allen & Ueno, 2018; Hitch et al., 2018). This boost does, however, typically come at a cost to the less valuable items presented in the same trial (e.g., Allen et al., 2024; Allen & Atkinson, 2021; Atkinson et al., 2018, 2024). Thus, prioritization instructions do not increase overall working memory capacity, but instead result in a re-allocation of resources toward the high value information (e.g., Allen et al., 2024; Atkinson et al., 2024; Hitch et al., 2018).

The prioritization effect is considered to result from the high value item being stored in an active and accessible privileged state, termed the focus of attention (e.g., Allen et al., 2024; Hu et al., 2014). The effect is robust and has been demonstrated across a wide variety of task contexts, including across different modalities (e.g., visual, verbal, and tactile information; Atkinson et al., 2021; Roe et al., 2024), presentation contexts (sequential and simultaneous presentation; Allen & Ueno, 2018; Atkinson et al., 2018; Hitch et al., 2018; Hu et al., 2014, 2016), and retrieval methods (cued-recall, recognition, and color reproduction tasks; Atkinson et al., 2022, 2024; Sandry et al., 2014, 2020). Whilst the majority of research has focused on testing young adults, prioritization effects have also been observed in older adults (Allen et al., 2021) and children (provided they are sufficiently motivated; Atkinson et al., 2019).

Studies to date have focused on neurotypical individuals. As such, it is not clear whether individuals with symptoms of ADHD, who often experience working memory difficulties, would be able to utilize the focus of attention within working memory to prioritize particularly valuable information. One possibility is that individuals with symptoms of ADHD may be less able to prioritize particularly valuable information in working memory. Supporting this possibility, there is some evidence that individuals with ADHD may have difficulties in orienting attentional resources during encoding (e.g., Kim et al., 2014). Moreover, Castel et al. (2011) found that some individuals with ADHD show less selectivity for valuable information during a long-term memory task. In this study, children with ADHD-Combined type (exhibiting difficulties with inattention and hyperactivity), ADHD-Inattentive type (exhibiting difficulties with inattention), and controls took part in a memory task. They were shown lists of 12 words to be remembered. Each word from the list was shown sequentially and paired with a number ranging from 1 to 12, indicating the number of points that it was worth. After each list had been presented, participants were asked to recall as many words from the list as possible within 30 s. They were told that the points collected could be exchanged for prizes at the end of the experiment. All groups reported more high value words relative to low value words. However, there was some evidence that children in the ADHD-Combined group were less selective relative to the other two groups. As such, it is possible that individuals with symptoms of ADHD would be less able to prioritize particularly valuable information in working memory.

A second possibility is that individuals with symptoms of ADHD may be able to direct attention in working memory as effectively as controls. Evidence for this is provided by Superbia-Guimarães et al. (2022), who tested 10- to 16-year-olds with ADHD and controls on a working memory task where participants had to recognize the color of animal drawings. Participants either received no location cues, were shown a pre-location cue (before the shapes appeared, an arrow pointed toward the location of the item that would later be tested), or a retro-cue (an arrow pointed at the relevant stimuli that would be tested after the animal shapes were shown). In this study, the ADHD and control groups benefitted equally from the pre-and retro-cues. Based on this it might be predicted that individuals with symptoms of ADHD will be able to direct their attention in working memory on the basis of reward as effectively as controls.

Finally, it is possible that individuals with symptoms of ADHD may show a larger prioritization boost relative to the controls. Indeed, there is some evidence that cognitive performance in individuals with ADHD may be more influenced by reward relative to matched controls (Dovis et al., 2012). Dovis et al. examined how motivational factors (such as monetary incentives) affect overall working memory performance of children with ADHD and controls. In their study, participants completed a visuo-spatial working memory task, under four conditions: feedback only, 1 euro, 10 euro, and “game.” In the feedback only condition, participants received only feedback about their performance. In the 1 euro and 10-euro conditions, participants were informed they could earn this amount of money if they performed well enough. The task was gamified in the “game” condition, with participants completing the same working memory task to progress a robot through a storyline. Children with ADHD performed better in the 1 euro, 10 euros, and game conditions relative to the feedback only condition. In contrast, there were no significant differences between the incentive conditions in controls. The incentives did not “normalize” performance in the ADHD group, however, with performance in the ADHD group significantly worse than the control group across all conditions. Taken together, this demonstrates that the effect of rewards can be greater for individuals with ADHD, but that the effects are not large enough to remove the working memory difficulties experienced in this group relative to controls. Nevertheless, based on these findings, it might be suggested that individuals with symptoms of ADHD will show a larger prioritization effect relative to controls.

We conducted a pair of experiments to investigate this. Participants were presented with sets of four colored shapes presented sequentially. After a short delay, the outline of each shape was presented (in a counterbalanced order), and participants were asked to report the color of the shape using colored buttons on screen. Participants completed two blocks of trials. In one block (equal value condition), they were told that all items were equally valuable, and that correct recall of each item would gain them two points. In the other block (differential value condition), participants were told that correct recall of the first item presented during encoding would gain them five points, whilst correct recall of the other items would gain them one point. In Experiment 1, feedback was presented on a trial-by-trial basis, informing participants which items they had responded correctly about, the number of points collected for each item, and the number of points collected in test trials overall so far. In Experiment 2, feedback was not presented, as is more typical in this paradigm (e.g., Allen & Ueno, 2018; Atkinson et al., 2018, 2021, 2022; Hu et al., 2014, 2016). Whilst participants completed the experiments, they engaged in articulatory suppression to disrupt verbal recoding of the visual information (Baddeley, 1986). This was to ensure that the task was indeed measuring visual working memory as intended, instead of participants also utilizing verbal working memory to retain the information. The experiments were conducted online, with participants recruited via Prolific. Participants in the ADHD-symptoms group considered themselves to have ADHD and reported symptoms consistent with a diagnosis on the Adult ADHD Self-Report Scale (ASRS; v1.1) Screener (e.g., Kessler et al., 2007). Meanwhile, those in the control group did not consider themselves to have ADHD and did not show symptoms consistent with a diagnosis on the ASRS Screener.

## Experiment 1

Experiment 1 investigated whether the ability to prioritize particularly valuable information in working memory differs in individuals with ADHD relative to controls without ADHD. A 2 (Value: Differential vs. Equal; within-subject) × 4 (Serial position [SP]: 1–4; within-subject) × 2 (Group: ADHD-symptoms vs. controls; between-subjects) mixed-design was employed. As in previous research, the value manipulation was targeted toward the first item (SP1; e.g., Atkinson et al., 2018, 2019). At SP1, it was expected that a significant effect of value would emerge, with participants exhibiting higher accuracy in the differential condition relative to the equal value condition. This would indicate that participants perform better at SP1 when this item is relatively more valuable than the other items in the trial, compared to a condition in which all items were as valuable as each other. Of particular interest was whether the value effect differed between groups. In particular, whether individuals with symptoms of ADHD would show increased, decreased, or similar sized prioritization effects relative to the control participants. At the less valuable SPs, it was expected that performance would be superior in the equal value condition relative to the differential value condition. This would indicate that prioritization of the particularly valuable item (SP1) comes at a cost to the other, less valuable serial positions (e.g., Allen et al., 2024; Allen & Atkinson, 2021; Atkinson et al., 2018, 2024). Again, of particular interest was whether the costs differed as a function of group.

### Method

#### Participants

Power analysis was conducted using G*Power (Faul et al., 2007). This was calculated for an ANOVA including value (within-subjects: differential vs. equal) and group (between-subjects: ADHD-symptoms vs. control) at SP1, since this is where the value manipulation was targeted. Based on a medium effect size of $\eta_{p}^{2}$ = .06 and alpha = .05, it was estimated that a total sample size of 54 participants across groups would provide 95% power. This sample size would also provide 95% power for an equivalent ANOVA (e.g., 2 (value [within-subjects]: differential vs. equal) × 2 (group [between-subjects]: ADHD-symptoms vs. control) on data averaged across the less valuable SPs (SPs 2–4).

All participants were recruited via Prolific. Eighty participants completed the experiment in total: 40 who considered themselves to have ADHD (ADHD-symptoms group) and 40 controls who did not consider themselves to have ADHD (control group). As we were interested in participants with symptoms of ADHD vs controls, we administered the ASRS screening measure for ADHD and excluded participants at the analysis stage whose degree of inattentive and hyperactive symptoms was inconsistent with the category to which they were assigned (i.e., we excluded participants in the ADHD-symptoms group who did not exhibit symptoms consistent with ADHD and participants in the control group who exhibited symptoms consistent with ADHD). Therefore, the ADHD-symptoms group reflects participants who consider themselves to have ADD/ADHD *and* have symptoms consistent with this diagnosis, whilst the Control group reflects participants who do not consider themselves to have ADD/ADHD *and* do not have symptoms consistent with an ADHD diagnosis (for a more detailed description of the recruitment approach, please see the Supplemental Materials). On this basis, three participants in the ADHD-symptoms group were excluded for scoring below the cut-off for ADHD on the ASRS screening measure, suggesting they do not exhibit behaviors highly consistent with ADHD. Meanwhile, eight participants in the control group were excluded for scoring above the cut-off for ADHD on the ASRS, suggesting these individuals were exhibiting behaviors highly consistent with ADHD. One control participant was then excluded due to a technical error with the audio recording, which prevented the articulatory suppression check being completed. Further, one control participant and one participant in the ADHD-symptoms group were excluded for not completing the articulatory suppression task as instructed. Finally, one control participant was excluded as their average performance across the task was below the chance guessing rate. The final sample therefore comprised 65 participants overall (36 in the ADHD-symptoms group: 17 female; 18 male; 1 non-binary; Mean [*M*_age_] = 27.92 years; standard deviation [*SD*] = 4.46; and 29 participants in the control group: 11 female; 18 male; *M*_age_ = 29.38, *SD* = 4.09). The groups did not significantly differ in terms of gender (*p* = .526) or age (*t*(63) = −1.36, *p* = .177). Sixteen participants in the ADHD-symptoms group had received a formal diagnosis of ADHD, whilst 19 had not and one preferred not to say. Seven participants in the ADHD-symptoms group reported taking medication for ADHD, whilst 29 responded that they were not taking medication for ADHD. All participants were recruited at a similar time of day and met the following criteria: 18 to 35 years of age, and had normal or corrected-to normal vision, no color blindness, English as a first language, no formal diagnosis of Autism or Dyslexia, resided in the UK, and an approval rate of 95% or higher on Prolific. Participants were paid £5 for their participation (a rate equivalent to £10/hr).

As expected, there was a significant difference between groups in the mean total ASRS Screener score (*t*(63) = 15.38, *p* < .001; range possible = 0–24), with the score higher in the ADHD-symptoms group (*M* = 18.36, *SD* = 2.54, Range = 14–24) than the control group (*M* = 8.21, *SD* = 2.77, Range = 1–13). The inattentive score (range possible: 0–16) was significantly higher in the ADHD-symptoms group (*M* = 12.39, *SD* = 2.03, Range = 9–16) than controls (*M* = 5.72, *SD* = 2.00 Range = 1–9; *t*(63) = 13.24, *p* < .001). The hyperactivity score (range possible: 0–8) was also significantly higher in the ADHD-symptoms group (*M* = 5.97, *SD* = 1.21, Range = 3–8) than the control group (*M* = 2.48, *SD* = 1.57, Range = 0–5; *t*(63) = 10.12, *p* < .001).

#### Materials

##### ASRS Screener

This comprises six items, designed to measure inattentive and hyperactive behaviors (e.g., delaying starting tasks and fidgeting). Participants report the extent to which they believe they have displayed each behavior in the last 6 months, selecting from the following options: “Never,” “Rarely,” “Sometimes,” “Often,” and “Very Often.” In line with our ethical approval, we also included a “Prefer not to say” option (although this option was not selected by any participants). The ASRS Screener is a commonly used tool, with high diagnostic accuracy internal consistency, and good test-re-test reliability (Brevik et al., 2020; Kessler et al., 2005, 2007; Lewczuk et al., 2024; Matza et al., 2011). Moreover, the 6-item ASRS screener used here is considered to perform at least as well as the original 18-item ASRS (Brevik et al., 2020; Kessler et al., 2005). In line with the ASRS Screener guidance, participants received a mark for each item if they responded: (i) “Sometimes,” “Often,” or “Very Often” to the first three items; and (ii) “Often” or “Very Often” to the final three items. Participants were recorded as scoring above the cut-off if they received four or more marks across the six items. This indicated the presence of attentional and hyperactive symptoms highly consistent with a diagnosis of ADHD. Meanwhile, participants were recorded as having not met the cut-off for ADHD if they received three or fewer marks. A total ASRS Screener score was also calculated per participant (Kessler et al., 2007). For each of the six statements, a numerical score was given (0 for “Never”; 1 for “Rarely”; “2” for “Sometimes”; 3 for “Often”; and 4 for “Very Often”). An overall score was calculated (minimum = 0, maximum = 24) which reflects the degree of inattentive and hyperactive symptoms (Kessler et al., 2007). Scores were also calculated for the total inattentive score (minimum = 0, maximum = 16) and the total hyperactivity score (minimum = 0, maximum = 8). For psychometric properties of the approaches used, we refer readers to Kessler et al. (2007).

##### Working Memory Task

In the working memory task, four items were presented sequentially in each trial. Stimuli were formed by pairing one of six shapes (circle, diamond, triangle, arrow, cross, and flag) with one of six colors (red, blue, green, yellow, magenta, and black). Pairings were formed with the constraint that no color or shape could be repeated within the same trial. Shapes (sized to fit within an imaginary 2 cm^2^) were presented on a white background. Locations were fixed, such that the items moved across the top of the screen from left to right (Allen et al., 2021). The four shapes were spaced equally about the horizontal center of the screen, separated by 4.3 cm horizontally, and located 1.8 cm above the vertical center of the screen. All items were tested on each trial in a counterbalanced order. The test probe was an outline of one of the shapes presented in the lower half of the screen with colored buttons underneath. The response buttons always appeared in the same order in a horizontal line (from left to right): red, blue, green, yellow, magenta, and black.

#### Design and Procedure

The study employed a 2 (value: differential vs. equal; within-subject) × 4 (SP: 1–4; within-subject) × 2 (Group: ADHD-symptoms vs. control; between-subject) mixed design. The dependent variable was accuracy (determined by the mean proportion of trials in which participants responded accurately). Participants completed two blocks of 24 trials: one for each “value” condition. The order of blocks was randomized. All SPs were tested on every trial, with the order of probes being counterbalanced within the value blocks and presented to participants in a random order. Thus, each SP was tested first, second, third, and fourth six times within each block. At the start of each block, participants completed four practice trials.

At the start of the experiment, participants completed a short questionnaire to collect demographic information, including age and gender, whether they have a formal diagnosis of ADHD, and whether they take any medication for ADHD. As part of this questionnaire, participants also completed the ASRS Screener (e.g., Kessler et al., 2007). There was then a calibration phase to ensure that the participants’ microphone was working correctly. In addition, participants were asked to re-size a rectangle presented on screen until it was the same size as a credit card. This enabled stimuli to be scaled based on the participants’ screen size. This is a standard approach which ensures that stimuli are presented at the same size across participants when conducting online experiments (Gresch et al., 2021, 2022; Li et al., 2020).

**Figure 1.**
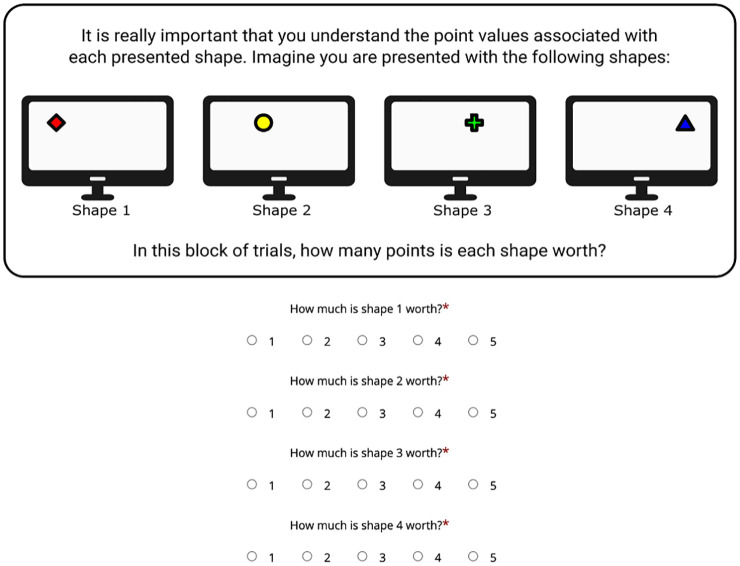
An example of how participants were required to demonstrate they had read and understood the instructions.

Each condition began with written instructions accompanied by pictorial representations. Participants were first given general instructions about the task. They were then asked to watch a short video of an example trial, which included a recording of the articulatory suppression at the correct rate (once per second). They were then told the point values associated with each item. In the differential value trial, participants were told that the first shape presented was worth 5 points and all other shapes were worth 1 point. In the equal value trial, they were told that each shape was worth 2 points. This reflected the number of points participants would collect if they responded correctly about each item. Points were notional and did not equate to any physical reward or monetary bonus. In each condition, participants were presented with an example set of shapes, which indicated how many points they would collect for each item. To ensure that instructions had been read and understood, participants were then presented with a visual example of stimuli and asked how many points each item was worth (see Figure 1). They needed to respond correctly about this to proceed. An incorrect response led to repetition of the instructions and the test question. This repeated until participants responded correctly.

The mean number of attempts the ADHD-symptoms group took to respond correctly was 1.00 in the equal condition (*SD* = 0.00) and 1.08 (*SD* = 0.28) in the differential value condition. Meanwhile, the mean number of times participants in the control group took to respond correctly was 1.10 (*SD* = 0.31) in the equal value condition and 1.17 (*SD* = 0.47) in the differential value condition (see Supplemental Material for frequencies). This did not significantly differ in either value condition (equal condition *p* = .084; differential condition *p* = .666).

**Figure 2.**
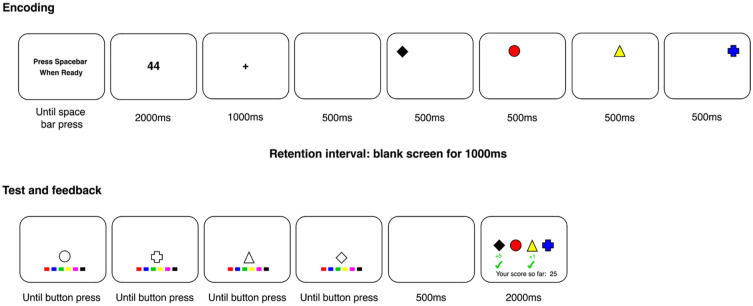
The experimental paradigm used in Experiment 1, with a differential value trial as an illustrative example. Figure not to scale.

The experimental paradigm is displayed in Figure 2. Each trial began with the on-screen message “Press Spacebar When Ready,” which remained on screen until participants pressed the spacebar. Next, a two-digit number pseudo-randomly selected between 20 and 99 was displayed on screen for 2,000 ms. Participants were asked to repeat this number aloud at a rate of one repetition a second to disrupt verbal rehearsal (Baddeley, 1986). This was followed by a fixation cross for 1,000 ms. After a blank interval of 500 ms, each shape was presented individually for 500 ms. There was then a 1,000 ms blank interval before all items were tested individually. At the test phase, each test probe remained on screen until the participant responded. After the participant had responded about all shapes, there was a 500 ms blank interval, before feedback was presented for 2,000 ms. This feedback screen informed participants which shapes they responded correctly about. The shapes were displayed in their correct colors in a horizontal line at the center of the screen in the same order as they were presented during the encoding phase. When the color of shapes had been correctly recalled, there was a green plus sign and number underneath (e.g., “+5”). This number corresponded to the number of points that participants had collected. A green tick was then presented underneath the number. Nothing was presented underneath shapes to which participants responded incorrectly. Underneath this was a running total, indicating the number of points participants had collected so far (e.g., “Your score so far: 25”). In practice blocks, this score started from 0 and was self-contained, so did not contribute toward their overall score. Their overall score started from 0 in the first test block, but carried over to the second test block, so that their performance in all test trials contributed toward their score at the end of the experiment.

To ensure compliance with the articulatory suppression instruction in the online testing environment, audio was recorded from the number being displayed until the end of the maintenance phase. Participants were told that their submission on Prolific would be rejected (i.e., they would not receive payment) if they did not engage in the articulatory suppression task as instructed. However, in reality, all participants were paid. Audio recordings were processed offline after the experiment to check compliance with the instruction.

Participants were given an untimed break halfway through each test block. The median time to complete the experiment was approximately 30 min.

#### Data Analysis

Data for both experiments and the analysis scripts are available on the Open Science Framework (OSF): https://osf.io/y3zw9/.

The main outcome measure was accuracy, determined as the proportion of correct responses. Frequentist analysis was conducted in R (R Core Team, 2023), using the afex (Singmann et al., 2023) and emmeans (Lenth, 2023) packages. Post-hoc comparisons for the frequentist ANOVAs were corrected using Bonferroni-Holm. Bayes Factor (BF) analysis was also conducted. This indicates the strength of evidence for the presence or absence of an effect. Bayesian ANOVAs were conducted using the “BayesFactor” package (Morey & Rouder, 2022) in R (R Core Team, 2023). Default priors were used, with 500,000 iterations. All models were calculated, meaning that a model could contain an interaction without the constituent main effects. In addition to reporting the best model, we report BFs for the individual main effects and interactions. These Bayes factors were computed by re-running the model with which_model set to “top.” This compares a model that omits a main effect/interaction to the model containing all main effects and interactions. This produces BF_01_ values, which indicates evidence of no effect. BF_10_ values were derived by inverting the (BF_01_) values (1/BF_01_). When BF_10_ is below 1, BF_01_ are also reported for ease of interpretation. BF_10_ above 3 were taken as evidence for an effect, whereas a BF_10_ below 0.33 (i.e., BF_01_ above 3) was taken as evidence of no effect. We primarily draw conclusions based on *p*-values, but we draw readers’ attention to any discrepancies that would result from interpreting *p*-values versus BFs.

The main analysis comprised a 2 (value; differential vs. equal; within-subject) × 2 (group: ADHD-symptoms vs. control; between-subject) mixed ANOVA at the targeted SP (SP1). A 2 (value; differential vs. equal; within-subject) × 2 (group: ADHD-symptoms vs control; between-subject) mixed ANOVA was also conducted on data averaged across the less valuable SPs (2–4).

Subsidiary analysis was also conducted to examine: (i) effects after excluding participants who reported taking medication for ADHD; and (ii) whether effects differ in individuals with and without a formal diagnosis of ADHD (see Supplemental Materials). To summarize, the conclusions obtained in all of these analyses did not differ from those reported in the main body below. We also conducted a three-way ANOVA (a 2 [value; differential vs. equal; within-subject] × 4 ]SP: 1–4] × 2 ]group: ADHD-symptoms vs. control; between-subject] ANOVA), to examine performance across all SPs within the same analysis and where costs to the less valuable SPs lie (see Supplemental Materials). We note that these outcomes should be interpreted with caution, however, as our power calculation was based on the main 2 (value; differential vs. equal; within-subject) × 2 (group: ADHD-symptoms vs. control; between-subject) mixed ANOVAs.

Finally, exploratory analysis was conducted to investigate whether there was a correlation between the ASRS Screener score, the prioritization boost at SP1 (i.e., performance in the differential condition at SP1 minus performance in the equal value condition), and the cost at the less valuable SPs (i.e., performance in the differential condition minus performance in the equal value condition). This analysis used the total ASRS Screener score, and thus reflected both inattentive and hyperactive behaviors. Higher scores on the ASRS reflect more symptoms of ADHD.

### Results

#### Effect at SP1 (Targeted SP)

**Figure 3.**
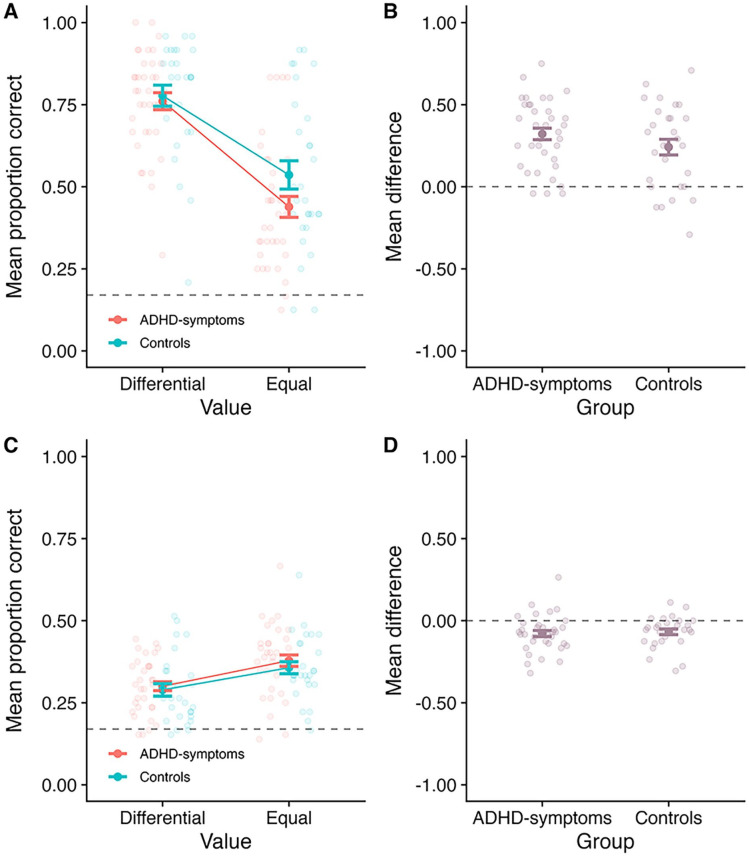
Outcomes for Experiment 1. Panel A: Mean proportion correct at SP1 as a function of value and group. Panel B: The mean difference between the differential and equal value conditions at SP1, as a function of group (calculating as Differential–Equal). Panel C: Mean proportion correct averaged across the less valuable SPs (SPs 2–4), as a function of value and group. Panel D: The mean difference between the differential and equal value conditions averaged across the less valuable SPs, as a function of group (calculating as Differential–Equal). In all panels, the bold point reflects the mean across participants, whilst error bars display standard error. The lighter points in the background reflect the means for individual participants. In Panels A and C, the dotted line at 0.17 reflects chance guessing rate, based on a stimulus set of six items. In Panels B and D, the dotted line at 0.00 reflects no difference.

Mean proportion correct at SP1 is displayed in Figure 3a, as a function of value and group. Meanwhile, Figure 3b displays the difference between the differential and equal value conditions as a function of group. A 2 (Value: differential vs. equal; within-subject) × 2 (Group: ADHD-symptoms vs. control; between-subjects) mixed ANOVA revealed a main effect of value at SP1 (*F*(1, 63 = 93.63, *MSE* = 0.03, *p* < .001, $\eta_{p}^{2}$ = .60; BF_10_ > 10,000) whereby participants exhibited higher performance in the differential value condition (*M* = 0.77, *SE* = 0.02) relative to the equal value condition (*M* = 0.49, *SE* = 0.03). There was no effect of group (*F*(1, 63) = 2.40, *MSE* = 0.04, *p* = .127, $\eta_{p}^{2}$ = .04; BF_10_ = 0.64, BF_01_ = 1.56), and no interaction between value and group (*F*(1, 63) = 1.91, *MSE* = 0.03, *p* = .172, $\eta_{p}^{2}$ = .03; BF_10_ = 0.60, BF_01_ = 1.66). We note that although the BF evidence for the interaction is not strongly in favor of no effect, the pattern (see Figure 3a and b) indicates a numerically larger effect in the ADHD-symptoms group than the control group. There is thus no evidence that the ability to prioritize valuable information is impaired in the ADHD-symptoms group relative to the control group. The BF analysis revealed the best model included a main effect of value (BF_10_ > 10,000 relative to the model containing only participant).

#### Effects Averaged Across Less Valuable SPs (2–4)

Mean proportion correct averaged across the less valuable SPs (2–4) is displayed in Figure 3c, as a function of value and group. Figure 3d shows the mean difference between the Differential and Equal value conditions as a function of group. A 2 (Value: differential vs. equal; within-subject) × 2 (Group: ADHD-symptoms vs. control; between-subjects) mixed ANOVA was conducted. There was a main effect of value (*F*(1, 63) = 31.50, *MSE* = 0.01, *p* < .001, $\eta_{p}^{2}$ = .33; BF_10_ > 10,000), with participants displaying better performance in the equal value condition (*M* = 0.37, *SE* = 0.01) relative to the differential value condition (*M* = 0.30, *SE* = 0.01). There was no effect of group (*F*(1, 63) = 0.66, *MSE* = 0.01, *p* = .421, $\eta_{p}^{2}$ = .01; BF_10_ = 0.37, BF_01_ = 2.73). There was also no interaction between value and group (*F*(1, 63) = 0.16, *MSE* = 0.01, *p* = .690, $\eta_{p}^{2}$ < .01; BF_10_ = 0.28, BF_01_ = 3.63). The BF analysis revealed the best model included a main effect of value (BF_10_ > 10,000 relative to the model containing only participant).

#### Correlations Between the Degree of Inattentive and Hyperactive Symptoms and Prioritization Boosts and Costs

**Figure 4.**
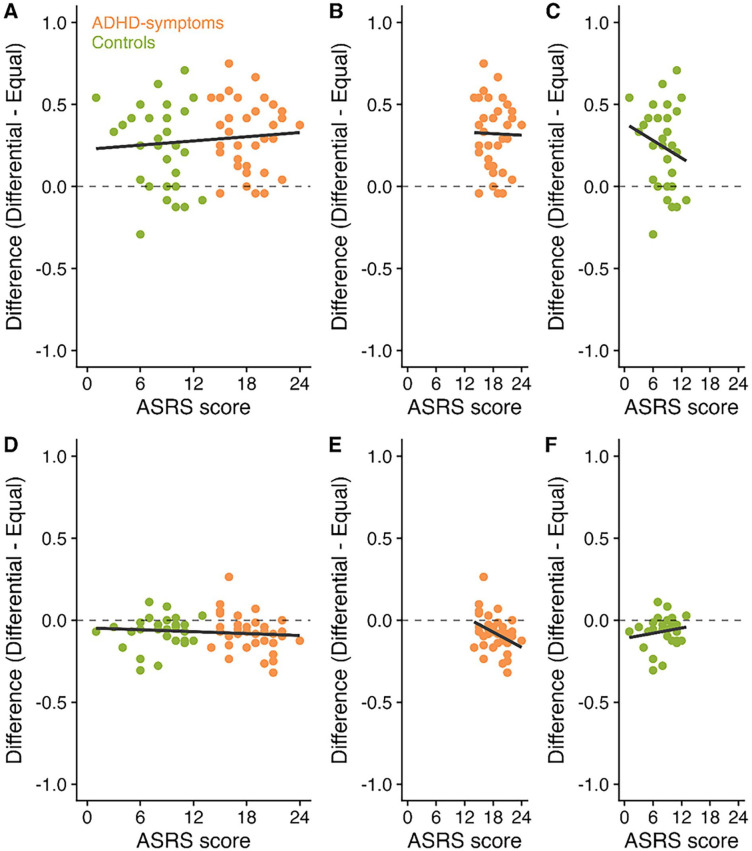
Correlations between ASRS Screener scores (reflecting the degree of inattentive and hyperactive behaviors), boosts to SP1 and costs to the less valuable items in Experiment 1. Panel A displays the correlation between ASRS Screener score and the difference in performance between the differential and equal value items at SP1 across participants. Panels B and C then present these correlations for the ADHD-symptoms and control groups, respectively. Panel D displays the correlation between ASRS Screener score and the difference in performance between the differential and equal value items at the less valuable SPs. Panels E and F then present these correlations for the ADHD-symptoms and control groups, respectively.

Exploratory analysis was then conducted to investigate whether the total ASRS Screener score (reflecting the degree of inattentive and hyperactive symptoms) correlates with the prioritization boost to SP1 and the cost to the other SPs. Figure 4 displays scatterplots of ASRS Screener scores, boosts to the high value item, and costs to the low value items.

Pearson’s correlation coefficients were conducted to investigate correlations between the ASRS Screener score (including all participants), the boost at SP1 and the costs at the less valuable SPs (see Table 1). Analysis was also conducted for each group separately. *p*-values were corrected using Bonferroni-Holm. Following correction, there was no significant correlation between any of the measures. In most cases, BF were at least anecdotally in favor of no correlations.^1^

**Table 1.** Correlations Between the ASRS Score, the Prioritization Boost at SP1 and the Prioritization Costs at the Less Valuable SPs (2–4) in Each Experiment.

Variables	*r*	*p*	BF_10_	BF_01_
Experiment 1				
ASRS score and prioritization boost at SP1 (all participants)	.10	1.000	0.39	2.59
ASRS score and prioritization cost at less valuable SPs (all participants)	−.11	1.000	0.39	2.53
ASRS score and prioritization boost at SP1 (ADHD-symptoms group)	−.02	1.000	0.37	2.69
ASRS score and prioritization cost at less valuable SPs (ADHD-symptoms group)	−.36	.197	2.68	0.37
ASRS score and prioritization boost at SP1 (Control group)	−.19	1.000	0.62	1.63
ASRS score and prioritization cost at less valuable SPs (Control group)	.16	1.000	0.53	1.87
Experiment 2				
ASRS score and prioritization boost at SP1 (all participants)	.05	1.000	0.30	3.39
ASRS score and prioritization cost at less valuable SPs (all participants)	<.01	1.000	0.28	3.62
ASRS score and prioritization boost at SP1 (ADHD-symptoms group)	.21	1.000	0.70	1.43
ASRS score and prioritization cost at less valuable SPs (ADHD-symptoms group)	−.26	.813	0.99	1.01
ASRS score and prioritization boost at SP1 (Control group)	.13	1.000	0.47	2.13
ASRS score and prioritization cost at less valuable SPs (Control group)	−.08	1.000	0.42	2.40

*Note. p*-Values corrected using Bonferroni-Holm within each experiment.

#### Discussion

This experiment was the first to investigate the extent to which individuals with symptoms of ADHD can direct their attention in working memory on the basis of value, and to compare this to a neurotypical group. Across groups, a prioritization boost was observed, whereby participants responded more accurately at the first SP when this item was associated with more notional points. Significant costs to the less valuable SPs were also observed. Importantly, there was no evidence that either the boost to SP1 or the costs to the less valuable SPs differed as a function of group. This provides the first evidence that individuals with symptoms of ADHD can prioritize information based on value in working memory, and that this ability is not impaired relative to controls.

These findings are broadly in line with those of Superbia-Guimarães et al (2022) who found that 10- to 16-year-olds with ADHD can direct their attention in working memory based on pre-cues and retro-cues as effectively as controls. The current findings extend this, demonstrating similar findings in adults and using an alternative type of attentional direction, considered to be distinct to predictive cue-based prioritization (Allen et al., 2024; Atkinson et al., 2018; Hitch et al., 2018). These observations run counter to existing findings suggesting that individuals with ADHD have difficulty allocating attention (Kim et al., 2014). Moreover, although the prioritization boost at SP1 was numerically larger in the ADHD-symptoms group, this was not supported by the inferential analyses. Therefore, these findings also do not align with empirical research suggesting that reward affects working memory performance to a greater extent in individuals with ADHD than controls (Dovis et al., 2012).

Evidence of costs of prioritization to less valuable items were observed. This fits with a large body of literature in neurotypical populations (e.g., Allen et al., 2024; Allen & Atkinson, 2021; Atkinson et al., 2018, 2024), suggesting that directing attention based on reward in working memory does not increase overall performance. Instead, it appears to result in a re-allocation of limited resources, whereby participants direct attention away from less valuable items (e.g., Allen et al., 2024; Atkinson et al., 2024; Hitch et al., 2018). As indicated in Figure 3 and the inferential analyses, the costs observed to the lower value items did not appear to differ between groups. This provides evidence that in individuals with symptoms of ADHD, prioritization similarly does not enhance overall capacity but instead results in a re-allocation of resources.

In the current experiment, participants were given feedback after every trial, informing them which items they responded correctly about, and the number of points collected for each item. This contrasts with much of the value-based prioritization literature, in which participants do not typically receive feedback at all (e.g., Allen et al., 2021; Allen & Ueno, 2018; Atkinson et al., 2018, 2021, 2022; Hu et al., 2014, 2016). The value effect observed in both groups in this experiment was considerably larger than that typically observed in previous studies, which may have resulted from the regular reinforcement of the point differences induced by the provision of feedback.

However, it is possible that the combination of value and feedback may not have impacted the groups to the same extent. Indeed, previous research has suggested that a combination of reward and feedback may particularly enhance working memory performance in individuals with ADHD (Hammer et al., 2015). In this study, boys with ADHD (*M*_age_ = 10.5 years) and age-matched controls (*M*_age_ = 10.9 years) completed a visual two-back task, in which they had to decide whether a letter presented on screen was in the same location as one presented two letters ago. The provision of trial-by-trial feedback (feedback vs. no feedback) and reward (large/small) was manipulated. In the large reward condition, the reward for correct responses was 10× larger than in the small reward condition. In the ADHD group, participants performed significantly better in the large-reward feedback condition relative to all other conditions. In contrast, controls performed significantly better in the large-reward no feedback condition relative to all other conditions. This suggests that the combination of feedback and reward may have been particularly beneficial for individuals with ADHD. Further supporting this, controls performed significantly better than the ADHD group in all conditions except the large-reward feedback condition, and the degree of ADHD symptoms predicted performance in all conditions except this one. Although this study involved offering rewards for task completion, rather than encouraging participants to direct attention toward particular items, it is possible that the absence of any group effects observed in the current experiment reflects the combination of value and trial-by-trial feedback employed. Thus, one possibility is that prioritization within working memory may be impaired in individuals with symptoms of ADHD relative to controls, when feedback is not provided (as is more typical in the value-based prioritization literature; Allen et al., 2021; Allen & Ueno, 2018; Atkinson et al., 2018, 2021, 2022; Hu et al., 2014, 2016). This was examined in Experiment 2.

## Experiment 2

Experiment 2 investigated whether adults with symptoms of ADHD would be able to direct attention in working memory based on reward as effectively as controls, when feedback was not provided. This is the paradigm more typically used when investigating this ability in neurotypical populations (Allen et al., 2021; Allen & Ueno, 2018; Atkinson et al., 2018, 2021, 2022; Hu et al., 2014, 2016). Participants completed the same visual working memory task as in Experiment 1, except that feedback was not provided at the end of each trial. Based on the outcomes from Experiment 1, it may be predicted that a value-based prioritization effect would be observed in the ADHD-symptoms group, with the magnitude of the effect a similar size to the control group. However, based on Hammer et al. (2015) it may be predicted that the prioritization effect may be smaller when trial-by-trial feedback is not provided.

### Method

#### Participants

The same recruitment approach was taken as in Experiment 1. The experiment was completed by 80 participants: 40 who consider themselves to have ADHD (ADHD-symptoms group) and 40 controls who do not consider themselves to have ADHD (control group). As in Experiment 1, participants where then excluded if their degree of inattentive and hyperactive symptoms (reported on the ASRS Screener) was inconsistent with the group to which they were assigned. On this basis, four participants in the control group were excluded for scoring above the cut-off for ADHD on the ASRS Screener, whilst two participants in the ADHD-symptoms group were excluded for scoring below the cut-off for ADHD on the ASRS Screener. A further four participants were excluded due to technical errors with the audio recording (three in the ADHD-symptoms group and one in the control group) which prevented the articulatory suppression from being checked. Finally, two participants (one in the ADHD-symptoms group and one in the control group) were excluded due to a failure to engage in the articulatory suppression task as instructed. No performance-based exclusions were applied, as all participants performed above chance guessing level. The final analysis was therefore performed on 34 individuals with symptoms of ADHD (19 female; 13 male; 2 non-binary, *M*_age_ = 26.35, *SD* = 4.17; M. years in education = 16.32; *SD* = 2.64) and 34 controls (14 female, 20 male; *M*_age_ = 29.97, *SD* = 3.97, M. years in education = 17.32; *SD* = 3.72). The groups did not significantly differ on gender (*p* = .132) or years in education (*t*(66) = −1.28, *p* = .206), but the ADHD-symptoms group were significantly younger than the control group (*t*(66) = −3.67, *p* < .001). In the ADHD-symptoms group, 11 had received a formal diagnosis of ADHD, 22 had no received a formal diagnosis, and one preferred not to say. Moreover, five participants in the ADHD-symptoms group reported taking medication for ADHD. As in Experiment 1, participants were recruited through Prolific, paid £5 for their participation (equivalent to £10/hr), and met the following criteria: 18 to 35 years of age, had normal or corrected-to normal vision, no color blindness, English as a first language, no formal diagnosis of Autism or Dyslexia, and resided in the UK. None of the participants had taken part in Experiment 1.

As expected, there was a significant difference between groups in the mean total ASRS Screener score (*t*(66) = 15.27, *p* < .001; range possible = 0–24), with the score higher in the ADHD-symptoms group (*M* = 18.41, *SD* = 2.75, Range = 13–24) than the control group (*M* = 7.12, *SD* = 3.32, Range = 0–12). The inattentive score (range possible: 0–16) was significantly higher in the ADHD-symptoms group (*M* = 12.21, *SD* = 2.06 Range = 9–16) than controls (*M* = 4.82, *SD* = 2.34, Range = 0–9, *t*(66) = 13.81, *p* < .001). The hyperactivity score (range possible: 0–8) was also significantly higher in the ADHD-symptoms group (*M* = 6.21, *SD* = 1.27, Range = 4–8) than the control group (*M* = 2.29, *SD* = 1.40, Range = 0–5; *t*(66) = 12.03, *p* < .001).

#### Materials, Design, and Procedure

The materials, design, and procedure were identical to Experiment 1, except that trial-by-trial feedback was not provided. To retain the same interval between retrieval and the initiation of the next trial the same as in Experiment 1, a blank screen was presented for 2,500 ms after participants responded about the last shape. A question was also included in the demographic information questionnaire to ask participants the number of years in education they had completed, in order to allow further demographic comparisons between the groups in this experiment.

Regarding the instructions check, the mean number of attempts the ADHD-symptoms group took to respond correctly was 1.09 in the equal condition (*SD* = 0.29) and 1.18 (*SD* = 0.39) in the differential value condition. Meanwhile, the mean number of times participants in the control group took to respond correctly was 1.12 (*SD* = 0.33) in the equal value condition and 1.03 (*SD* = 0.17) in the differential value condition (see Supplemental Material for frequencies). There were no significant differences between groups in either condition (equal value condition *p* = 1.00; differential value condition *p* = .105).

### Results

#### Effect at SP1 (Targeted SP)

**Figure 5.**
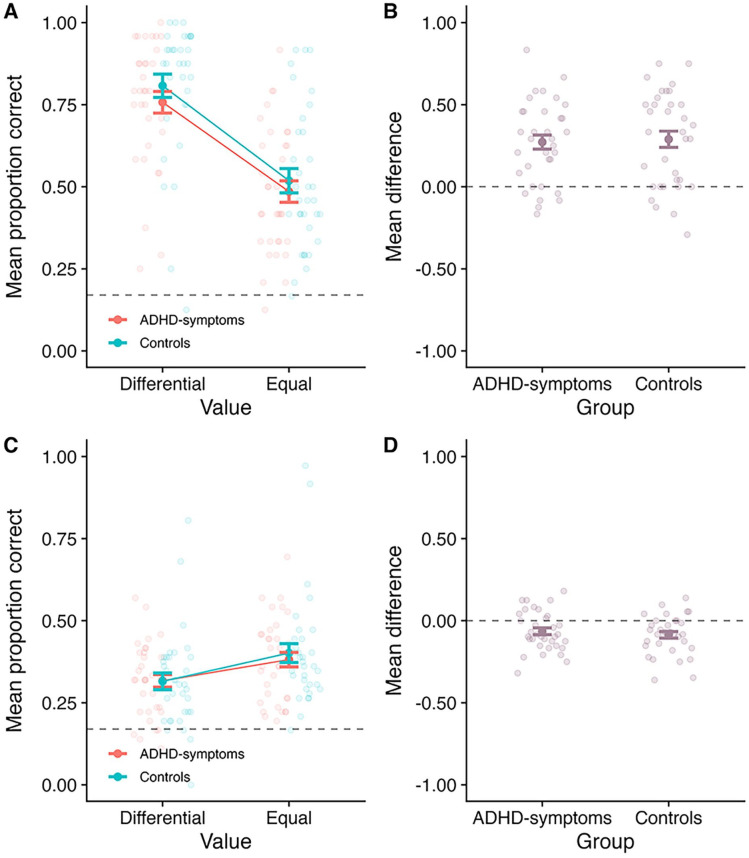
Outcomes for Experiment 2. Panel A: Mean proportion correct at SP1 as a function of value and group. Panel B: The mean difference between the differential and equal value conditions at SP1, as a function of group (calculating as Differential–Equal). Panel C: Mean proportion correct averaged across the less valuable SPs, as a function of value and group. Panel D: The mean difference between the differential and equal value conditions averaged across the less valuable SPs, as a function of group (calculating as Differential–Equal). In all panels, the bold point reflects the mean across participants, whilst error bars display standard error. The lighter points in the background reflect the means for individual participants. In Panels A and C, the dotted line at 0.17 reflects chance guessing rate, based on a stimulus set of six items. In Panels B and D, the dotted line at 0.00 reflects no difference.

Mean proportion correct at SP1 is displayed in Figure 5a, as a function of value and group. Figure 5b displays the difference between the differential and equal value conditions as a function of group. A 2 (Value: differential vs. equal; within-subject) × 2 (Group: ADHD-symptoms vs. control; between-subjects) mixed ANOVA revealed a significant main effect of value (*F*(1, 66) = 73.49, *MSE* = 0.04, *p* < .001, $\eta_{p}^{2}$ = .53; BF_10_ > 10,000) whereby participants exhibited higher performance in the differential value condition (*M* = 0.78, *SE* = 0.02). relative to the equal value condition (*M* = 0.50, *SE* = 0.02). There was no effect of group (*F*(1, 66) = 1.31, *MSE* = 0.05, *p* = .256, $\eta_{p}^{2}$ = .02; BF_10_ = 0.37, BF_01_ = 2.68), and no interaction between value and group (*F*(1, 66) = 0.07, *MSE* = 0.04, *p* = .794, $\eta_{p}^{2}$ < .01; BF_10_ = 0.26, BF_01_ = 3.90). BF analysis revealed that the best model included a main effect of value (BF_10_ > 10,000 relative to the model containing participant only).

#### Effects Averaged Across Less Valuable SPs (2–4)

Mean proportion correct averaged across the less valuable SPs (2–4) is displayed in Figure 5c, as a function of value and group. Meanwhile, Figure 5d displays the mean difference between the Differential and Equal value conditions as a function of group. A 2 (Value: differential vs. equal; within-subject) × 2 (Group: ADHD-symptoms vs. control; between-subjects) mixed ANOVA found a significant main effect of value (*F*(1, 66) = 27.33, *MSE* = 0.01, *p* < .001, $\eta_{p}^{2}$ = .29; BF_10_ = 9,387.86) whereby participants performed better in the equal value condition (*M* = 0.39, *SE* = 0.02) relative to the differential value condition (*M* = 0.32, *SE* = 0.02). There was no effect of group (*F*(1, 66) = 0.09, *MSE* = 0.03, *p* = .767, $\eta_{p}^{2}$ < .01; BF_10_ = 0.36, BF_01_ = 2.81), and no interaction between value and group (*F*(1, 66) = 0.56, *MSE* = 0.01, *p* = .455, $\eta_{p}^{2}$ = .01; BF_10_ = 0.31, BF_01_ = 3.19). The BF analysis revealed that the best model included a main effect of value (BF_10_ = 7,734.59 relative to the model containing participant only).

#### Correlations Between the Degree of Inattentive and Hyperactive Symptoms and Prioritization Boosts and Costs

**Figure 6.**
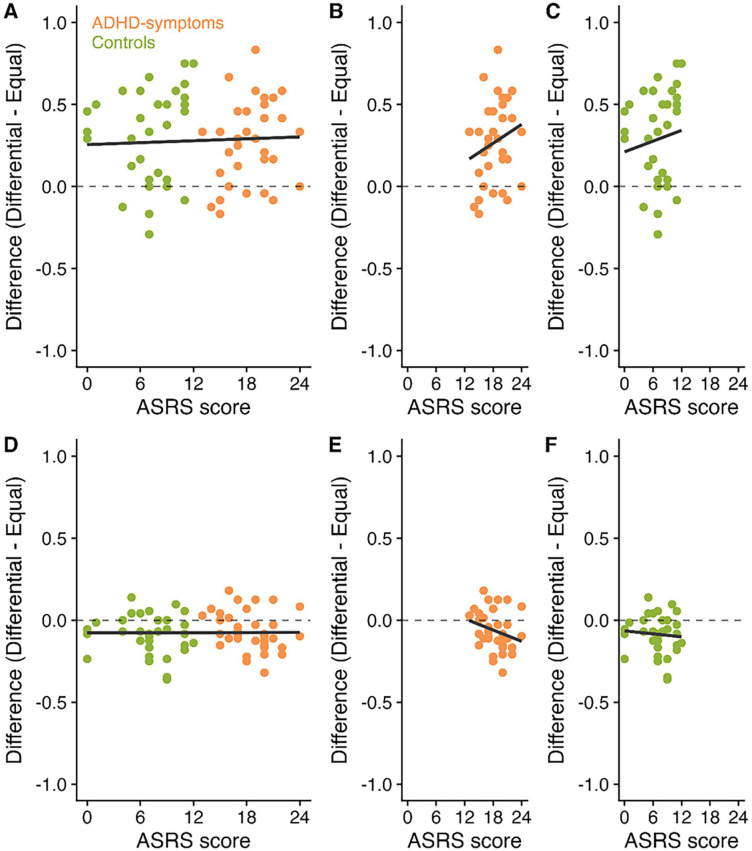
Correlations between ASRS Screener scores (reflecting the degree of inattentive and hyperactive behaviors), boosts to SP1 and costs to the less valuable items in Experiment 2. Panel A displays the correlation between ASRS Screener score and the difference in performance between the differential and equal value items at SP1 across participants. Panels B and C then present these correlations for the ADHD-symptoms and control groups, respectively. Panel D displays the correlation between ASRS Screener score and the difference in performance between the differential and equal value items at the less valuable serial positions. Panels E and F then present these correlations for the ADHD-symptoms and control groups, respectively.

As in Experiment 1, exploratory analysis was then conducted to investigate whether the degree of inattentive and hyperactive symptoms correlates with the prioritization boost (at SP1) and the cost (at the other SPs). Figure 6 displays scatterplots of ASRS Screener scores, boosts to the high value item, and costs to the low value items. Pearson’s correlation coefficients were conducted to investigate correlations between scores on the ASRS Screener and the prioritization boosts at SP1 and the costs at the less valuable SPs. *p*-Values were corrected using Bonferroni-Holm. There was no significant correlation between the ASRS Screener score and the prioritization boost, either when considering all participants or each group separately (see Table 1). In all cases, the BFs were at least anecdotally in favor of no correlations (although the BF for the correlation between the ASRS Screener score and the cost to less valuable SPs in the ADHD-symptoms group was close to 1; refer note 1).

#### Discussion

Previous research has suggested that the combination of reward and feedback may be particularly beneficial for working memory performance in individuals with ADHD (Hammer et al., 2015). Experiment 2 therefore investigated whether prioritization boosts could be observed in individuals with symptoms of ADHD in the absence of trial-by-trial feedback. The results demonstrated that individuals with ADHD were able to prioritize a particularly high value item in working memory in the absence of trial-by-trial feedback. Further, there were no interactions including group, demonstrating that the effects observed did not significantly differ between the ADHD-symptoms group and a control group. These findings are in line with Experiment 1, providing further evidence that individuals with symptoms of ADHD do not exhibit difficulties in value-based prioritization within working memory. Further, these findings indicate that the ability to prioritize particularly valuable information in individuals with symptoms of ADHD is not contingent on trial-by-trial reinforcement regarding the points system. As in Experiment 1, there were costs to the less valuable SPs, with no evidence that these differed across groups. This provides further support for the notion that individuals with symptoms of ADHD experience costs of prioritization in a similar way to controls.

## Cross-Experimental Analyses

Given the similarities between the experiments, cross-experimental analyses were conducted to investigate whether effects differed as a function of a group when the data for both experiments was combined, thus providing greater statistical power. This analysis therefore ignores experiment (i.e., whether trial-by-trial feedback was provided or not).

### Performance at SP1

**Figure 7.**
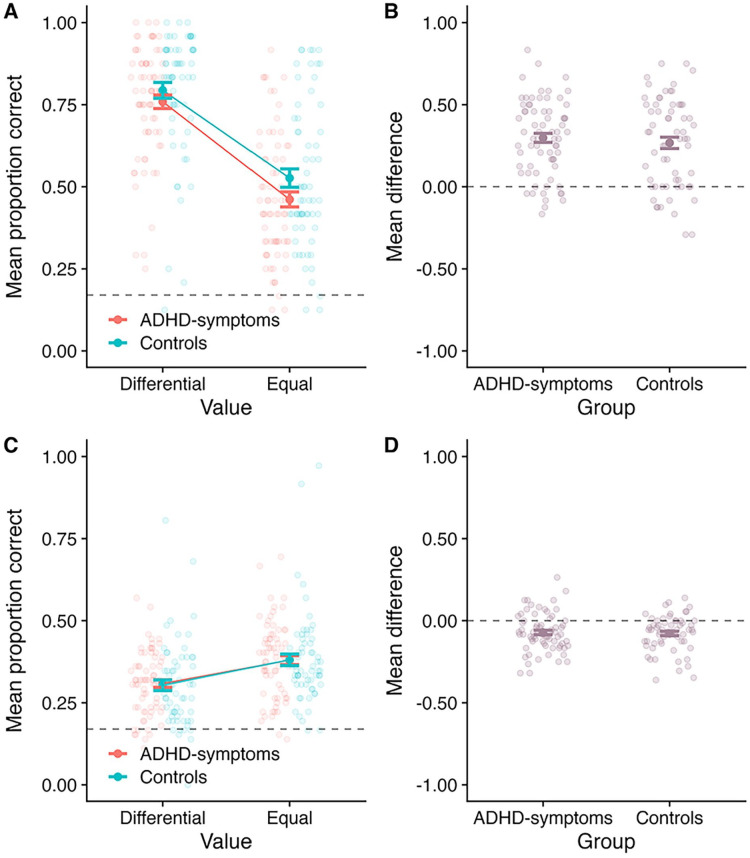
Outcomes when data for both experiments were combined. Panel A: Mean proportion correct at SP1 as a function of value and group. Panel B: The mean difference between the differential and equal value conditions at SP1, as a function of group (calculating as Differential–Equal). Panel C: Mean proportion correct averaged across the less valuable SPs, as a function of value and group. Panel D: The mean difference between the differential and equal value conditions averaged across the less valuable SPs, as a function of group (calculating as Differential–Equal). In all panels, the bold point reflects the mean across participants, whilst error bars display standard error. The lighter points in the background reflect the means for individual participants. In Panels A and C, the dotted line at 0.17 reflects chance guessing rate, based on a stimulus set of six items. In Panels B and D, the dotted line at 0.00 reflects no difference.

Mean performance at SP1 is displayed in Figure 7a, as a function of value and group. Figure 7b displays the difference between the differential and equal value conditions as a function of group. A 2 (Value: differential vs. equal; within-subject) × 2 (Group: ADHD-symptoms vs. control; between-subjects) mixed ANOVA was conducted. This revealed a significant main effect of value (*F*(1, 131) = 166.64, *MSE* = 0.03, *p* < .001, $\eta_{p}^{2}$ = .56; BF_10_ > 10,000) with higher accuracy observed in the differential value condition (*M* = 0.78, *SE* = 0.02). relative to the equal value condition (*M* = 0.49, *SE* = 0.02). There was no effect of group (*F*(1, 131) = 3.77, *MSE* = 0.04, *p* = .054, $\eta_{p}^{2}$ = .03; BF_10_ = 0.85, BF_01_ = 1.17), and no interaction between value and group (*F*(1, 131) = 0.48, *MSE* = 0.03, *p* = .488, $\eta_{p}^{2}$ < .01; BF_10_ = 0.23, BF_01_ = 4.27). BF analysis revealed that the best model included a main effect of value (BF_10_ > 10,000 relative to the model containing participant only).

### Effects Averaged Across Less Valuable SPs (2–4)

Mean performance the less valuable SPs is presented in Figure 7c, as a function of value and group. Figure 7d presents the difference between the differential and equal value conditions as a function of group. A 2 (Value: differential vs. equal; within-subject) × 2 (Group: ADHD-symptoms vs. control; between-subjects) mixed ANOVA revealed a significant main effect of value (*F*(1, 131) = 59.51, *MSE* = 0.01, *p* < .001, $\eta_{p}^{2}$ = .31; BF_10_ > 10,000) with participants performing more accurately in the equal value condition (*M* = 0.38, *SE* = 0.01) relative to the differential value condition (*M* = 0.31, *SE* = 0.01). No effect of group emerged (*F*(1, 131) = 0.01, *MSE* = 0.02, *p* = .911, $\eta_{p}^{2}$ < .01; BF_10_ = 0.25, BF_01_ = 4.00), and there was also no interaction between value and group (*F*(1, 131) = 0.10, *MSE* = 0.01, *p* = .750, $\eta_{p}^{2}$ < .01; BF_10_ = 0.20, BF_01_ = 5.10). BF analysis revealed that the best model included a main effect of value (BF_10_ > 10,000 relative to the model containing participant only).

### Other Cross-Experimental Analyses

Analysis was also performed to investigate whether correlations emerged between ASRS Screener scores, the prioritization boost at SP1, and the prioritization costs across all participants (ignoring experiment). Following correction for multiple comparisons, there were no significant correlations (although the BF analysis did provide some support for a correlation between the ASRS Screener and costs to the less valuable SPs in the ADHD-symptoms group only; see Supplemental Materials for full reporting).

Furthermore, analysis was performed across all data in both experiments to investigate whether the effects observed differ depending on whether individuals had a formal diagnosis of ADHD or not. The effects were similar across all groups (Control, ADHD-symptoms group (with formal diagnosis), ADHD-symptoms group (without formal diagnosis; see Supplemental Materials for full reporting).

Finally, analysis was conducted to investigate whether overall performance and the size of the prioritization effects differed between experiments, where trial-by-trial feedback was present (Experiment 1) and absent (Experiment 2). Given that the above analyses found no effect of group and that the prioritization effects did not differ as a function of group, the effects were examined across group to maximize statistical power. The outcomes are presented in the Supplemental Materials. To summarize, there was no effect of Experiment, and no interactions including Experiment. This provides evidence that the provision of trial-by-trial feedback did not impact overall performance, or the size of the prioritization effects.

## General Discussion

The current pair of experiments investigated the extent to which adults with symptoms of ADHD can direct their attention in working memory to particularly valuable information, and whether this ability was impaired (or enhanced) relative to individuals without symptoms of ADHD. The provision of trial-by-trial feedback was varied across experiments, with feedback provided in Experiment 1 and absent in Experiment 2. Across both experiments, performance for the high value item (SP1) was superior in the differential value condition, relative to the equal value condition. As in previous research, there were also costs to the less valuable items. Crucially, there was no evidence that the boost to the particularly valuable item or the costs to the less valuable items differed as a function of group. This provides evidence that adults with symptoms of ADHD were as able to direct attention in working memory based on value as control participants. Moreover, exploratory analyses revealed no consistent evidence that the degree of inattentive and hyperactive symptoms (measured by the ASRS Screener) correlated with prioritization effects, either across groups or within the groups. Similar outcomes were also observed across a number of subsidiary and cross-experimental analyses (see Supplemental Materials), indicating that the findings are highly robust to alternative, plausible analytical decisions. Taken together, these experiments provide the first evidence that individuals with symptoms of ADHD can prioritize particularly valuable information in working memory, with no impairment observed relative to control participants.

Evidence that individuals with ADHD symptoms are as able to direct attention in working memory is in line with previous research which found similar findings using predictive cueing paradigms with children and adolescents (Superbia-Guimarães et al., 2022). Taken together, these findings suggest that the ability to use the focus of attention within working memory to focus on particularly valuable or task-relevant information is not impaired in individuals with symptoms of ADHD. In the current pair of experiments, the boost at the first (targeted) serial position was accompanied by costs to less valuable items. Within non-clinical groups, this has been taken as evidence that value instructions do not enhance participants’ working memory capacity, but instead results from a re-distribution of attention, whereby participants focus more on the targeted item at the expense of others in the sequence (e.g., Allen et al., 2024; Atkinson et al., 2024; Hitch et al., 2018). Here, a similar pattern was observed across both groups, suggesting a similar pattern is present in individuals with symptoms of ADHD and controls. Thus, increasing the value of a particular item did not appear to motivate participants with symptoms of ADHD to simply try harder or perform better on the task in general. Instead, as with controls, it resulted in them focusing their existing working memory resources toward the particularly valuable information.

Previous studies with non-clinical groups suggested that prioritization effects are likely to emerge, in part, through participants refreshing the high value item more during the retention interval (Atkinson et al., 2022). Given that the values associated with items are known at encoding, these effects may also at least partially reflect participants directing their attention toward the item during this phase (Allen et al., 2024). Whilst the current pair of experiments suggest that individuals with symptoms of ADHD are as able to direct attention in working memory as controls, underlying mechanisms were not explored. As such, it is possible that the mechanisms underlying the prioritization effects observed differ in individuals with symptoms of ADHD and controls. Some existing research may support this possibility. For example, previous studies have found that individuals with ADHD have difficulties allocating attention during the encoding phase (e.g., Kim et al., 2014). As such, it may be that individuals with symptoms of ADHD achieve the same size prioritization boost as controls, but through different strategic approaches. It would be beneficial for further research to investigate this.

Previous research has found that overall working memory accuracy is enhanced in children and adolescents with ADHD if participants have the potential to earn financial rewards. For example, Dovis et al. (2012) found that individuals with ADHD performed better on a working memory task if they had the potential to earn 1 euro or 10 euros, relative to a condition which only feedback was provided. In contrast, controls performed no differently in these conditions. The current findings are somewhat inconsistent with this, finding that increasing the value of particular items did not result in a greater boost for individuals with symptoms of ADHD. These differences may reflect differences between the studies. For example, it is possible that the effect of value was not larger in the ADHD-symptoms group relative to the control group due to the rewards offered. In the current pair of experiments, participants could earn notional points. However, in previous research finding larger effects in the ADHD-symptoms group, participants were able to earn financial reward. Studies in neurotypical adults have found that the size of prioritization effects in working memory do not substantially differ when using notional rewards or financial incentives (Zheng et al., 2022). However, it is possible that the potential to earn financial incentives have a larger impact in the ADHD-symptoms group. A second possibility is that the difference may reflect the age groups of the participants tested. Adults were tested in the current pair of experiments, compared to children and adolescents in Dovis et al. (2012). Therefore, one possibility is that the effect of value may become smaller over time in individuals with ADHD.

There was no overall effect of group in either experiment. This suggests that individuals with symptoms of ADHD performed no worse than controls in these experiments. Given that working memory difficulties are commonly observed in individuals with ADHD, it is possible that there could have been a selection bias whereby participants in the ADHD-symptoms group may have less impaired working memory relative to the ADHD population as a whole. However, we believe this is unlikely to be the case for several reasons. Firstly, all participants included in the ADHD-symptoms group screened positively for ADHD on the ASRS Screener, indicating that they exhibited symptoms that were highly consistent with ADHD. Secondly, the outcomes did not differ in individuals with and without a formal diagnosis of ADHD (see Supplemental Materials). Finally, there was no significant correlation between the ASRS Screener score, the prioritization boost at the first serial position, and the prioritization cost at the less valuable serial positions. Therefore, there is no evidence to suggest that the effects observed differ depending on the degree of inattentive and hyperactive symptoms. An alternative possibility for the absence of a group effect is that the ADHD-symptoms group may have been more motivated when completing the task in general relative to the control group, possibly due to the potential to earn points. If so, this may mask any overall performance differences between the groups. However, this is unlikely to explain the absence of an effect, as previous research has found that even financial rewards do not “normalize” working memory performance in individuals with ADHD (Dovis et al., 2012). As such, it is unlikely that the potential to earn notional points (with no real-world value) would eliminate any differences between groups.

A related possibility is that the ADHD-symptoms group may have impairments in the ability to direct attention in working memory, but that these were masked because the ADHD-symptoms group tried harder to prioritize the high value item. However, this is unlikely due to several reasons. Firstly, if participants in the ADHD-symptoms group were trying harder to direct attention to SP1, this should have been accompanied by greater costs to the less valuable items. This was not observed in either experiment or in the cross-experimental comparisons. Secondly, if this was the case, one might have expected that the ADHD-symptoms group would show a smaller prioritization effect in Experiment 2 when they received no feedback regarding the points system. However, the results of both experiments were consistent in showing no impairment in the ADHD-symptoms group’s ability to direct attention in working memory. Thirdly, evidence that the ADHD-symptoms group showed no impairment in their ability to direct attention in working memory is consistent with existing research using other manipulations that do not involve the manipulation of points or rewards (e.g., cueing; Superbia-Guimarães et al., 2022). As such, this explanation is unlikely to explain the pattern of results observed.

The cross-experimental analyses revealed no difference in overall performance between the experiments, and that the prioritization effects observed did not differ as a function of experiment. This suggests that prioritization effects in working memory are not significantly affected by whether trial-by-trial feedback is provided. This adds to a growing body of research identifying factors that do, and do not, appear to affect prioritization effects in working memory. For example, whilst the number of items presented does appear to influence the size of the effect (Atkinson et al., 2019; Zheng et al., 2022), neither the type of reward (e.g., notional points vs. monetary rewards; Zheng et al., 2022) or the provision of trial-by-trial feedback appears to.

In both experiments, the value manipulation was targeted toward SP1. This was based on previous research, which have often targeted the first SP when implementing this paradigm (e.g., Atkinson et al., 2018, 2019). As we directly compared data between the differential and an equal value condition at SP1, our comparison would not be confounded by any primacy effect which is typically observed in working memory tasks (higher accuracy often observed at the first SP). Within non-clinical adult samples, there is evidence that individuals can prioritize valuable information presented at early, middle, or late positions in a sequence (Atkinson et al., 2021; Hitch et al., 2018). It is, however, possible that prioritization at different SPs may require different skills or abilities. For example, if the first item is associated with a higher value, individuals may need to protect the privileged status associated with this item when encoding subsequent items. The need to do this may be greatly reduced if participants are asked to prioritize later positions. However, prioritizing these later positions may instead require other skills (e.g., tracking the position of items within the sequence). It would therefore be beneficial for further research to investigate prioritization in individuals with symptoms of ADHD at other positions within the sequence. It would also be beneficial for further research to examine prioritization in individuals with ADHD in other contexts. For example, research with non-clinical adult samples has begun to explore the extent to which individuals can direct attention in working memory when value information is provided only during the retention interval (Allen & Atkinson, 2021; Jeanneret et al., 2023, 2024; Hautekiet et al., 2024). This may be more challenging, as individuals cannot direct attention during the encoding phase in this paradigm (Allen & Atkinson, 2021; see Allen et al., 2024). Indeed, prioritization effects are markedly smaller in such tasks than when individuals can prioritize information during encoding (Allen & Atkinson, 2021). One possibility is that individuals with symptoms of ADHD would show impairments in the ability to direct attention in working memory under these more challenging circumstances.

Although our primary conclusions were drawn based on the outcomes from the ANOVAs, we also conducted exploratory analyses examining correlations between the ASRS, boosts to SP1, and costs to the less valuable SPs. However, it is worth highlighting that the ASRS was primarily implemented in these experiments as a screening tool to aid group assignment and the range was somewhat limited (i.e., on a 0–24 scale). It is therefore possible that correlations between the degree of inattentive and hyperactive symptoms and the prioritization effects may be observed if a more fine-grained measure of ADHD symptoms was used. It would be beneficial for further research to investigate this.

A further limitation is that the groups were not age-matched in Experiment 2, with the ADHD-symptoms group significantly younger than the control group. However, it is unlikely this affected overall performance between the groups, as working memory abilities are relatively stable between the ages of 18 to 35 years (the possible age range of participants; Brockmole & Logie, 2013). This is also unlikely to have affected the size of the prioritization effects observed, as the ability to prioritize valuable information in working memory is preserved even in older adulthood (Allen et al., 2021). Moreover, it is possible that the groups could have differed in an unmeasured variable that could explain the pattern of results observed (e.g., education and/or IQ in Experiment 1, or IQ in Experiment 2). Although there is no evidence that the ability to direct attention in working memory is linked to factors such as educational level or IQ, the absence of such measures remains a limitation of this study. It may therefore be beneficial for future research to further examine whether the ability to direct attention in working memory is impaired in individuals with ADHD who are matched on a wider range of variables (e.g., age, gender, education level, and IQ).

To summarize, across all analyses, there was clear evidence that individuals with symptoms of ADHD are able to prioritize particularly valuable information in working memory. The ability in this group did not significantly differ relative to controls, suggesting no impairment in the ability to prioritize particularly valuable information in working memory. Converging evidence of this was also demonstrated from correlational analyses, which found no significant associations between scores on the ASRS Screener (indicating the degree of inattentive and hyperactive symptoms) and the prioritization boost at the targeted SP (SP1) and costs at the less valuable serial positions. Taken together, this study provides clear and consistent evidence that the ability to prioritize particularly valuable information in working memory is not impaired in individuals with symptoms of ADHD.

## Supplemental Material

sj-docx-1-jad-10.1177_10870547251330039 – Supplemental material for The Ability to Direct Attention in Working Memory Is Not Impaired in Adults With Symptoms of ADHD Supplemental material, sj-docx-1-jad-10.1177_10870547251330039 for The Ability to Direct Attention in Working Memory Is Not Impaired in Adults With Symptoms of ADHD by Amy L. Atkinson, Beatriz Pinheiro Sanchez, Matthew Warburton, Heather Allmark and Richard J. Allen in Journal of Attention Disorders

## Notes

1. There were also no significant correlations between the ASRS, boosts at SP1 and costs to the less valuable items following Bonferroni-Holm correction when measures of inattention and hyperactivity were analysed separately.

## References

[bibr1-10870547251330039] Adult ADHD Self-Report Scale (ASRS; v1.1) Screener. Retrieved August 19, 2024, from https://add.org/wp-content/uploads/2015/03/adhd-questionnaire-ASRS111.pdf

[bibr2-10870547251330039] Alderson R. M. Kasper L. J. Hudec K. L. Patros C. H. (2013). Attention-deficit/hyperactivity disorder (ADHD) and working memory in adults: A meta-analytic review. Neuropsychology, 27(3), 287–302. 23688211 10.1037/a0032371

[bibr3-10870547251330039] Allen R. Atkinson A. (2021). Retrospective and prospective prioritization in visual working memory. PsyArXiv. 10.31234/osf.io/4x8zu

[bibr4-10870547251330039] Allen R. J. Atkinson A. Hitch G. J. (2024). Getting value out of working memory through strategic prioritisation; Implications for storage and control. Quarterly Journal of Experimental Psychology, 78(2), 405–424. 10.1177/17470218241258102 PMC11783991 38769883

[bibr5-10870547251330039] Allen R. J. Atkinson A. L. Nicholls L. A. B. (2021). Strategic prioritisation enhances young and older adults’ visual feature binding in working memory. Quarterly Journal of Experimental Psychology, 74(2), 363–376. 10.1177/1747021820960712 PMC8044628 32933421

[bibr6-10870547251330039] Allen R. J. Ueno T. (2018). Multiple high-reward items can be prioritized in working memory but with greater vulnerability to interference. Attention, Perception, & Psychophysics, 80, 1731–1743. 10.3758/s13414-018-1543-6 29968084

[bibr7-10870547251330039] Alloway T. P. (2006). How does working memory work in the classroom? Educational Research and Reviews, 1(4), 134–139.

[bibr8-10870547251330039] Atkinson A. L. Allen R. J. Baddeley A. D. Hitch G. J. Waterman A. H. (2021). Can valuable information be prioritized in verbal working memory? Journal of Experimental Psychology: Learning, Memory, and Cognition, 47(5), 747–764. 33136420 10.1037/xlm0000979

[bibr9-10870547251330039] Atkinson A. L. Berry E. D. Waterman A. H. Baddeley A. D. Hitch G. J. Allen R. J. (2018). Are there multiple ways to direct attention in working memory? Annals of the New York Academy of Sciences, 1424(1), 115–126. 29635690 10.1111/nyas.13634 PMC6849770

[bibr10-10870547251330039] Atkinson A. L. Oberauer K. Allen R. J. Souza A. S. (2022). Why does the probe value effect emerge in working memory? Examining the biased attentional refreshing account. Psychonomic Bulletin & Review, 29(3), 891–900. 35091995 10.3758/s13423-022-02056-6 PMC9166884

[bibr11-10870547251330039] Atkinson A. L. Waterman A. H. Allen R. J. (2019). Can children prioritize more valuable information in working memory? An exploration into the effects of motivation and memory load. Developmental Psychology, 55(5), 967–980. 30816725 10.1037/dev0000692

[bibr12-10870547251330039] Atkinson A. L. Waterman A. H. Allen R. J. (2024). Does value-based prioritization at working memory enhance long-term memory? Memory & Cognition, 52, 1983–1998. 38378883 10.3758/s13421-024-01532-9 PMC11588910

[bibr13-10870547251330039] Baddeley A. D. (1986). Working memory. Oxford University Press, Clarendon Press.

[bibr14-10870547251330039] Baddeley A. D. Hitch G. J. Allen R. J. (2021). A multicomponent model of working memory. In Logie R. H. Camos V. Cowan N. (Eds.), Working memory: State of the science (pp. 10–43). Oxford University Press.

[bibr15-10870547251330039] Brevik E. J. Lundervold A. J. Haavik J. Posserud M. B. (2020). Validity and accuracy of the adult attention-deficit/hyperactivity disorder (ADHD) self-report scale (ASRS) and the Wender Utah rating scale (WURS) symptom checklists in discriminating between adults with and without ADHD. Brain and Behavior, 10(6), e01605. 10.1002/brb3.1605 PMC7303368 32285644

[bibr16-10870547251330039] Brockmole J. R. Logie R. H. (2013). Age-related change in visual working memory: A study of 55,753 participants aged 8–75. Frontiers in Psychology, 4, 12. 23372556 10.3389/fpsyg.2013.00012 PMC3557412

[bibr17-10870547251330039] Cain K. Oakhill J. Bryant P. (2004). Children’s reading comprehension ability: Concurrent prediction by working memory, verbal ability, and component skills. Journal of Educational Psychology, 96(1), 31–42.

[bibr18-10870547251330039] Castel A. D. Lee S. S. Humphreys K. L. Moore A. N. (2011). Memory capacity, selective control, and value-directed remembering in children with and without attention-deficit/hyperactivity disorder (ADHD). Neuropsychology, 25(1), 15–24. 20873928 10.1037/a0020298 PMC6615734

[bibr19-10870547251330039] Cowan N. (2014). Working memory underpins cognitive development, learning, and education. Educational Psychology Review, 26, 197–223. 25346585 10.1007/s10648-013-9246-y PMC4207727

[bibr20-10870547251330039] Cowan N. (2017). The many faces of working memory and short-term storage. Psychonomic Bulletin & Review, 24, 1158–1170. 27896630 10.3758/s13423-016-1191-6

[bibr21-10870547251330039] Dovis S. Van der Oord S. Wiers R. W. Prins P. J. (2012). Can motivation normalize working memory and task persistence in children with attention-deficit/hyperactivity disorder? The effects of money and computer-gaming. Journal of Abnormal Child Psychology, 40, 669–681. 22187093 10.1007/s10802-011-9601-8 PMC3375007

[bibr22-10870547251330039] Faraone S. V. Biederman J. Mick E. (2006). The age-dependent decline of attention deficit hyperactivity disorder: A meta-analysis of follow-up studies. Psychological Medicine, 36(2), 159–165. 16420712 10.1017/S003329170500471X

[bibr23-10870547251330039] Faul F. Erdfelder E. Lang A. G. Buchner A. (2007). G* Power 3: A flexible statistical power analysis program for the social, behavioral, and biomedical sciences. Behavior Research Methods, 39(2), 175–191. 17695343 10.3758/bf03193146

[bibr24-10870547251330039] Fürst A. J. Hitch G. J. (2000). Separate roles for executive and phonological components of working memory in mental arithmetic. Memory & Cognition, 28, 774–782. 10983451 10.3758/bf03198412

[bibr25-10870547251330039] Gathercole S. E. Lamont E. M. I. L. Y. Alloway T. P. (2006). Working memory in the classroom. In Pickering S. J. (Ed.), Working memory and education (pp. 219–240). Academic Press.

[bibr26-10870547251330039] Gresch D. Boettcher S. E. Nobre A. C. van Ede F. (2022). Consequences of predictable temporal structure in multi-task situations. Cognition, 225, Article 105156. 10.1016/j.cognition.2022.105156 PMC9760566 35537346

[bibr27-10870547251330039] Gresch D. Boettcher S. E. Van Ede F. Nobre A. C. (2021). Shielding working-memory representations from temporally predictable external interference. Cognition, 217, Article 104915. 10.1016/j.cognition.2021.104915 PMC8543071 34600356

[bibr28-10870547251330039] Hammer R. Tennekoon M. Cooke G. E. Gayda J. Stein M. A. Booth J. R. (2015). Feedback associated with expectation for larger-reward improves visuospatial working memory performances in children with ADHD. Developmental Cognitive Neuroscience, 14, 38–49. 26142072 10.1016/j.dcn.2015.06.002 PMC4536089

[bibr29-10870547251330039] Hautekiet C. Langerock N. Vergauwe E. (2024). Prioritization in visual working memory: An investigation of distractor susceptibility and different prioritization modes. PsyArXiv. https://osf.io/ae9y4 10.1037/xlm0001534 41021527

[bibr30-10870547251330039] Hitch G. J. Hu Y. Allen R. J. Baddeley A. D. (2018). Competition for the focus of attention in visual working memory: Perceptual recency versus executive control. Annals of the New York Academy of Sciences, 1424(1), 64–75. 29524359 10.1111/nyas.13631

[bibr31-10870547251330039] Hu Y. Allen R. J. Baddeley A. D. Hitch G. J. (2016). Executive control of stimulus-driven and goal-directed attention in visual working memory. Attention, Perception, & Psychophysics, 78, 2164–2175. 10.3758/s13414-016-1106-7 27142524

[bibr32-10870547251330039] Hu Y. Hitch G. J. Baddeley A. D. Zhang M. Allen R. J. (2014). Executive and perceptual attention play different roles in visual working memory: Evidence from suffix and strategy effects. Journal of Experimental Psychology: Human Perception and Performance, 40(4), 1665–1678. 24933616 10.1037/a0037163

[bibr33-10870547251330039] Jeanneret S. Bartsch L. M. Vergauwe E. (2023). To be or not to be relevant: Comparing short-and long-term consequences across working memory prioritization procedures. Attention, Perception, & Psychophysics, 85(5), 1486–1498. 10.3758/s13414-023-02706-4 PMC10151114 37127814

[bibr34-10870547251330039] Jeanneret S. Vergauwe E. Hautekiet C. Langerock N. (2024). What are the benefits of directed attention within verbal working memory? Quarterly Journal of Experimental Psychology, 78(2), 337–369. 10.1177/17470218241299918 PMC11783979 39501579

[bibr35-10870547251330039] Kasper L. J. Alderson R. M. Hudec K. L. (2012). Moderators of working memory deficits in children with attention-deficit/hyperactivity disorder (ADHD): A meta-analytic review. Clinical Psychology Review, 32(7), 605–617. 22917740 10.1016/j.cpr.2012.07.001

[bibr36-10870547251330039] Kessler R. C. Adler L. Ames M. Demler O. Faraone S. Hiripi E. V. A. Howes M. J. Jin R. Secnik K. Spencer T. Ustun T. B. Walters E. E. (2005). The World Health Organization Adult ADHD Self-Report Scale (ASRS): A short screening scale for use in the general population. Psychological Medicine, 35(2), 245–256. 15841682 10.1017/s0033291704002892

[bibr37-10870547251330039] Kessler R. C. Adler L. Barkley R. Biederman J. Conners C. K. Demler O. Faraone S. V. Greenhill L. L. Howes M. J. Secnik K. Spencer T. Ustun T. B. Walters E. E. Zaslavsky A. M. (2006). The prevalence and correlates of adult ADHD in the United States: Results from the National Comorbidity Survey Replication. American Journal of Psychiatry, 163(4), 716–723. 16585449 10.1176/appi.ajp.163.4.716 PMC2859678

[bibr38-10870547251330039] Kessler R. C. Adler L. A. Gruber M. J. Sarawate C. A. Spencer T. Van Brunt D. L. (2007). Validity of the World Health Organization Adult ADHD Self-Report Scale (ASRS) Screener in a representative sample of health plan members. International Journal of Methods in Psychiatric Research, 16(2), 52–65. 17623385 10.1002/mpr.208 PMC2044504

[bibr39-10870547251330039] Kim S. Liu Z. Glizer D. Tannock R. Woltering S. (2014). Adult ADHD and working memory: Neural evidence of impaired encoding. Clinical Neurophysiology, 125(8), 1596–1603. 24411642 10.1016/j.clinph.2013.12.094

[bibr40-10870547251330039] Kofler M. J. Rapport M. D. Bolden J. Altro T. A. (2008). Working memory as a core deficit in ADHD: Preliminary findings and implications. The ADHD Report, 16(6), 8–14.

[bibr41-10870547251330039] Lenth R. (2023). emmeans: Estimated Marginal Means, aka Least-Squares Means_. R package version 1.8.8. https://CRAN.R-project.org/package=emmeans

[bibr42-10870547251330039] Lewczuk K. Marcowski P. Wizła M. Gola M. Nagy L. Koós M. Kraus S. W. Demetrovics Z. Potenza M. N. Ballester-Arnal R. Batthyány D. Bergeron S. Billieux J. Briken P. Burkauskas J. Cárdenas-López G. Carvalho J. Castro-Calvo J. Chen L. . . . Bőthe B. (2024). Cross-cultural adult ADHD assessment in 42 countries using the adult ADHD self-report scale screener. Journal of Attention Disorders, 28(4), 512–530. 38180045 10.1177/10870547231215518

[bibr43-10870547251330039] Li Q. Joo S. J. Yeatman J. D. Reinecke K. (2020). Controlling for participants’ viewing distance in large-scale, psychophysical online experiments using a virtual chinrest. Scientific Reports, 10(1), 904. 31969579 10.1038/s41598-019-57204-1 PMC6976612

[bibr44-10870547251330039] Martinussen R. Hayden J. Hogg-Johnson S. Tannock R. (2005). A meta-analysis of working memory impairments in children with attention-deficit/hyperactivity disorder. Journal of the American Academy of Child & Adolescent Psychiatry, 44(4), 377–384. 15782085 10.1097/01.chi.0000153228.72591.73

[bibr45-10870547251330039] Matza L. S. Van Brunt D. L. Cates C. Murray L. T. (2011). Test–retest reliability of two patient-report measures for use in adults with ADHD. Journal of Attention Disorders, 15(7), 557–563. 20837987 10.1177/1087054710372488

[bibr46-10870547251330039] Morey R. Rouder J. (2022). BayesFactor: Computation of bayes factors for common designs. R package version 0.9.12-4.4. https://CRAN.R-project.org/package=BayesFactor

[bibr47-10870547251330039] R Core Team. (2023). R: A language and environment for statistical computing. R Foundation for Statistical Computing. https://www.R-project.org

[bibr48-10870547251330039] Ramos A. A. Hamdan A. C. Machado L. (2020). A meta-analysis on verbal working memory in children and adolescents with ADHD. The Clinical Neuropsychologist, 34(5), 873–898. 31007130 10.1080/13854046.2019.1604998

[bibr49-10870547251330039] Rapport M. D. Bolden J. Kofler M. J. Sarver D. E. Raiker J. S. Alderson R. M. (2009). Hyperactivity in boys with attention-deficit/hyperactivity disorder (ADHD): A ubiquitous core symptom or manifestation of working memory deficits? Journal of Abnormal Child Psychology, 37, 521–534. 19083090 10.1007/s10802-008-9287-8

[bibr50-10870547251330039] Roe D. Allen R. J. Elsley J. Miles C. Johnson A. J. (2024). Working memory prioritisation effects in tactile immediate serial recall. Quarterly Journal of Experimental Psychology, 77(11), 2354–2363. 10.1177/17470218241231283 PMC11529111 38282209

[bibr51-10870547251330039] Sandry J. Schwark J. D. MacDonald J. (2014). Flexibility within working memory and the focus of attention for sequential verbal information does not depend on active maintenance. Memory & Cognition, 42, 1130–1142. 24879637 10.3758/s13421-014-0422-1

[bibr52-10870547251330039] Sandry J. Zuppichini M. D. Ricker T. J. (2020). Attentional flexibility and prioritization improves long-term memory. Acta Psychologica, 208, Article 103104. 10.1016/j.actpsy.2020.103104 32622150

[bibr53-10870547251330039] Sibley M. H. Mitchell J. T. Becker S. P. (2016). Method of adult diagnosis influences estimated persistence of childhood ADHD: A systematic review of longitudinal studies. The Lancet Psychiatry, 3(12), 1157–1165. 27745869 10.1016/S2215-0366(16)30190-0

[bibr54-10870547251330039] Sibley M. H. Swanson J. M. Arnold L. E. Hechtman L. T. Owens E. B. Stehli A. Abikoff H. Hinshaw S. P. Molina B. S. G. Mitchell J. T. Jensen P. S. Howard A. L. Lakes K. D. Stern K. (2017). Defining ADHD symptom persistence in adulthood: Optimizing sensitivity and specificity. Journal of Child Psychology and Psychiatry, 58(6), 655–662. 27642116 10.1111/jcpp.12620 PMC5809153

[bibr55-10870547251330039] Simone A. N. Marks D. J. Bédard A. C. Halperin J. M. (2018). Low working memory rather than ADHD symptoms predicts poor academic achievement in school-aged children. Journal of Abnormal Child Psychology, 46, 277–290. 28357519 10.1007/s10802-017-0288-3 PMC5620112

[bibr56-10870547251330039] Singmann H. Bolker B. Westfall J. Aust F. Ben-Shachar M. (2023). afex: Analysis of factorial experiments. R package version 1.3-0. https://CRAN.R-project.org/package=afex

[bibr57-10870547251330039] Smith-Spark J. H. Fisk J. E. (2007). Working memory functioning in developmental dyslexia. Memory, 15(1), 34–56. 17479923 10.1080/09658210601043384

[bibr58-10870547251330039] Souza A. S. Oberauer K. (2016). In search of the focus of attention in working memory: 13 years of the retro-cue effect. Attention, Perception, & Psychophysics, 78, 1839–1860. 10.3758/s13414-016-1108-5 27098647

[bibr59-10870547251330039] Superbia-Guimarães L. Bader M. Camos V. (2022). Attentional orienting in working memory in children with ADHD. Developmental Neuropsychology, 47(8), 384–400. 36514838 10.1080/87565641.2022.2155164

[bibr60-10870547251330039] Thomas R. Sanders S. Doust J. Beller E. Glasziou P. (2015). Prevalence of attention-deficit/hyperactivity disorder: A systematic review and meta-analysis. Pediatrics, 135(4), e994–e1001. 10.1542/peds.2014-3482 25733754

[bibr61-10870547251330039] Waterman A. H. Atkinson A. L. Aslam S. S. Holmes J. Jaroslawska A. Allen R. J. (2017). Do actions speak louder than words? Examining children’s ability to follow instructions. Memory & Cognition, 45, 877–890. 28315065 10.3758/s13421-017-0702-7 PMC5529483

[bibr62-10870547251330039] Yang T. X. Allen R. J. Holmes J. Chan R. C. (2017). Impaired memory for instructions in children with attention-deficit hyperactivity disorder is improved by action at presentation and recall. Frontiers in Psychology, 8, 39. 28174550 10.3389/fpsyg.2017.00039 PMC5258743

[bibr63-10870547251330039] Zheng W. Geng J. Zhang D. Zhang J. Qiao J. (2022). Task difficulty rather than reward method modulates the reward boosts in visual working memory. Journal of Vision, 22(11), 1. 10.1167/jov.22.11.1 PMC9547356 36194408

