# Supplementary material for: The Ability to Direct Attention in Working Memory Is Not Impaired in Adults With Symptoms of ADHD
Source: J Atten Disord. 2025 Apr 18;29(9):684–705. doi: 10.1177/10870547251330039 (PMC12145475; doi:10.1177/10870547251330039)
Supplement: sj-docx-1-jad-10.1177_10870547251330039 – Supplemental material for The Ability to Direct Attention in Working Memory Is Not Impaired in Adults With Symptoms of ADHD [file sj-docx-1-jad-10.1177_10870547251330039.docx]

**Supplementary materials for: “The ability to direct attention in working memory is not impaired in adults with symptoms of attention deficit hyperactivity disorder (ADHD)”.**

| **1** | Further details about the recruitment approach |
| --- | --- |
| **2** | Attempts at instructions questions in Experiments 1 and 2 |
| **3** | Excluding participants receiving medication for ADHD (Experiment 1) |
| **4** | Investigating effects by ADHD diagnosis (Experiment 1) |
| **5** | Excluding participants receiving medication for ADHD (Experiment 2) |
| **6** | Investigating effects by ADHD diagnosis (Experiment 2) |
| **7** | Correlations between the ASRS Screener and the prioritization boosts and costs (across experiments) |
| **8** | Investigating effects by ADHD diagnosis (across experiments) |
| **9** | Three-way ANOVAs |
| **10** | Investigating the effects of trial-by-trial feedback by comparing the experiments |

1. **Further details about the recruitment approach**

All participants were recruited from Prolific at similar times of day. All participants who took part in the study had been asked by Prolific whether they “consider themselves to have attention deficit disorder (ADD)/attention deficit hyperactivity disorder (s)”. Participants who responded “Yes” or “No” were eligible to take part in this study (assuming they met the other inclusion criteria – e.g. based on age, colour blindness, etc). Participants who responded “Yes” were assigned to the ADHD-symptoms group, whilst participants who responded “No” were assigned to the Control group. As part of our demographic questionnaire, we asked participants this question ourselves to ensure their response matched what they had reported to Prolific. If their respond did not match the response they had given to Prolific, they were screened out of the study and replaced. To ensure that our ADHD-symptoms and Control groups included participants with and without ADHD symptoms respectively, participants completed the ASRS Screener. At the analysis stage, participants in the ADHD-symptoms group were excluded if their responses on the ASRS Screener suggested they did not exhibit behaviours consistent with ADHD. Meanwhile, participants in the Control group were excluded if their responses on the ASRS suggested they had symptoms consistent with ADHD. Therefore, the ADHD-symptoms group reflects participants who consider themselves to have ADD/ADHD *and* have symptoms consistent with this diagnosis, whilst the Control group reflects participants who do not consider themselves to have ADD/ADHD *and* do not have symptoms consistent with an ADHD diagnosis.

All participants in both groups were required to meet the following criteria: 18-35 years of age, and had normal or corrected-to normal vision, no colour blindness, English as a first language, no formal diagnosis of Autism or Dyslexia, and resided in the UK. Participants were explicitly asked these questions as part of the demographic questionnaire, and screened out and replaced if they did not meet these criteria. Participants were also required to have a Prolific approval rating of ≥ 95%.

1. **Attempts at instructions questions in Experiments 1 and 2**

**Experiment 1:**

| *Table S.2.1.* The number of attempts required to pass the instructions, as a function of group and value condition in Experiment 1. | | |
| --- | --- | --- |
|  | Group | |
|  | ADHD-symptoms  (*N* = 36) | Control  (*N* = 29) |
| Equal |  |  |
| 1 attempt | 36 (100%) | 26 (89.66%) |
| 2 attempts | 0 (0%) | 3 (10.34%) |
| 3 attempts | 0 (0%) | 0 (0%) |
| Differential |  |  |
| 1 attempt | 33 (91.67%) | 25 (86.21%) |
| 2 attempts | 3 (8.33%) | 3 (10.34%) |
| 3 attempts | 0 (0%) | 1 (3.45%) |

**Experiment 2:**

| *Table S.2.2.* The number of attempts required to pass the instructions, as a function of group and value condition in Experiment 2. | | |
| --- | --- | --- |
|  | Group | |
|  | ADHD-symptoms  (*N* = 36) | Control  (*N* = 29) |
| Equal |  |  |
| 1 attempt | 31 (91.18%) | 30 (88.24%) |
| 2 attempts | 3 (8.82%) | 4 (11.76%) |
| Differential |  |  |
| 1 attempt | 28 (82.35%) | 33 (97.06%) |
| 2 attempts | 6 (17.65%) | 1 (2.94%) |

1. **Excluding participants receiving medication for ADHD (Experiment 1)**

Seven participants in the ADHD-symptoms group reported taking medication for ADHD. Subsidiary analysis was therefore conducted to investigate whether the pattern of results presented in the Results section differed when participants receiving medication for ADHD were excluded. This analysis was conducted on data from 58 participants (29 in the ADHD-symptoms group and 29 in the control group). The groups did not significantly differ on age (*p* = .226) or gender (*p* = .292). In the ADHD-symptoms group, nine had a formal diagnosis, 19 had no formal diagnosis, and one preferred not to say.

*Performance at SP1*

Mean proportion correct at SP1 is plotted in Figure S.3.1.A as a function of value and group. This analysis revealed a significant main effect of value (*p* < .001), whereby participants performed more accurately in the differential value condition (*M* = 0.77, *SE* = 0.02) relative to the equal value condition (*M* = 0.50, *SE* = 0.03). There was no significant effect of group (*p* = .324), and no interaction between value and group (*p* = .313).

*
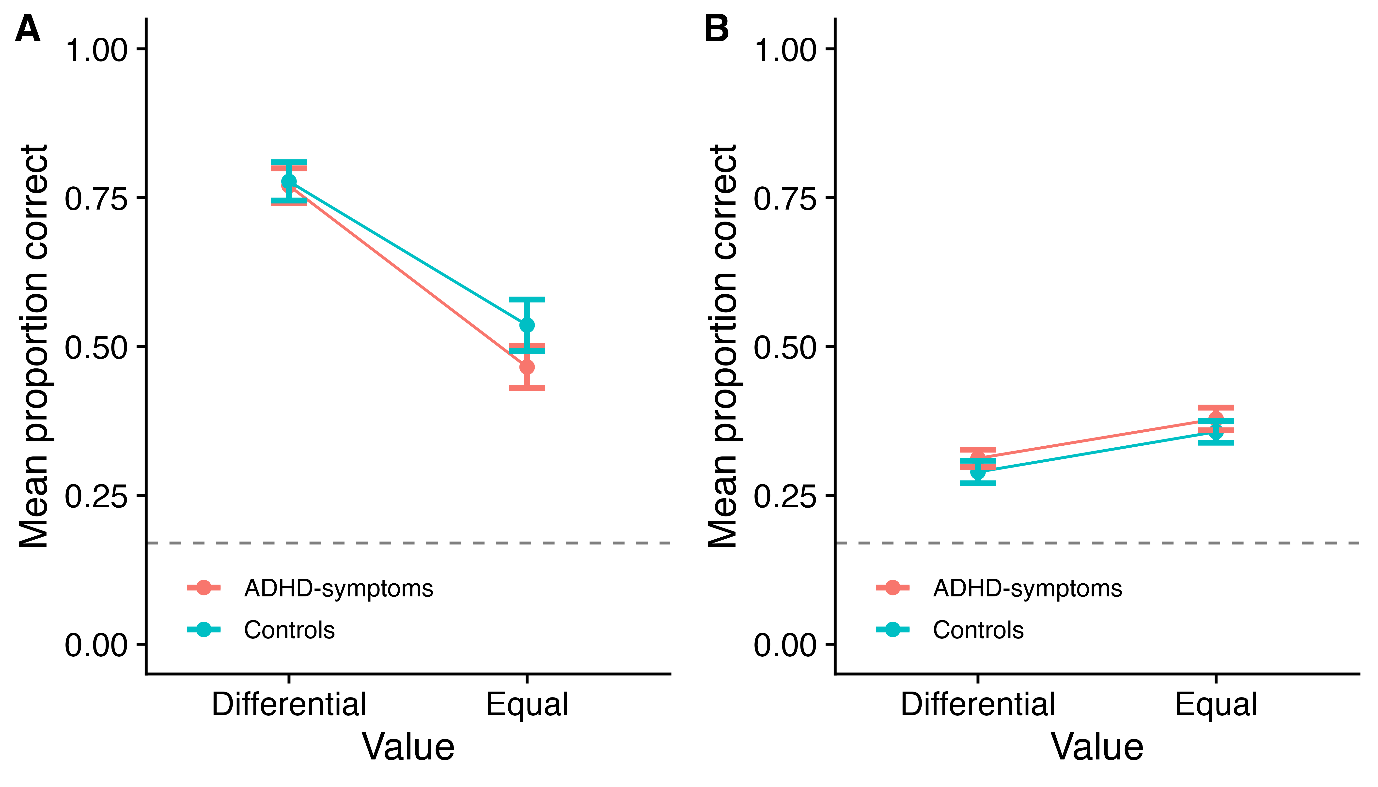
Figure S.3.1.* Task performance excluding participants taking medication for ADHD. Panel A: Mean proportion correct at SP1, as a function of value and group. Panel B: Mean proportion correct at the less valuable SPs, as a function of value and group. Error bars show standard error. The dotted line at 0.17 indicates chance guessing rate.

*Performance at the less valuable SPs*

Mean proportion correct at the less valuable SPs is plotted in Figure S.3.1.B as a function of value and group. There was a significant main effect of value (*p* < .001), whereby accuracy was higher in the equal value condition (*M* = 0.37, *SE* = 0.01) relative to the differential value condition (*M* = 0.30, *SE* = 0.01). There was no effect of group (*p* = .301), and no interaction between value and group (*p* = .957).

Taken together, this demonstrates that the main conclusions of Experiment 1 not differ when excluding participants who were taking medication for ADHD at the time of testing.

1. **Investigating effects by ADHD diagnosis (Experiment 1)**

Descriptive statistics were computed to investigate whether the pattern of results differed depending on whether participants in the ADHD-symptoms group had a formal diagnosis of ADHD or not. This plot included all participants included in the main body of the manuscript, except one participant in the ADHD-symptoms group who were excluded as they responded “rather not to say” to the question asking whether they had a formal diagnosis of ADHD. Mean proportion correct at SP1 is shown in Figure S.4.1.A, as a function of value and group (Control group, ADHD-symptoms (Formal diagnosis), ADHD-symptoms (No formal diagnosis)). Meanwhile, mean proportion correct at the less valuable SPs is shown in Figure S.4.1.B, as a function of value and group (Control group, ADHD-symptoms (Formal diagnosis), ADHD-symptoms (No formal diagnosis)). Although inferential statistics were not performed due to the small sample sizes for the ADHD-symptoms subgroups (*N* = 16 for the formal diagnosis subgroup and N = 19 for the no formal diagnosis subgroup), it is clear that all groups show the broadly same pattern, with a prioritization boost at SP1. Inferential analysis was conducted on the data across both experiments to examine this further (see supplementary materials section 8).


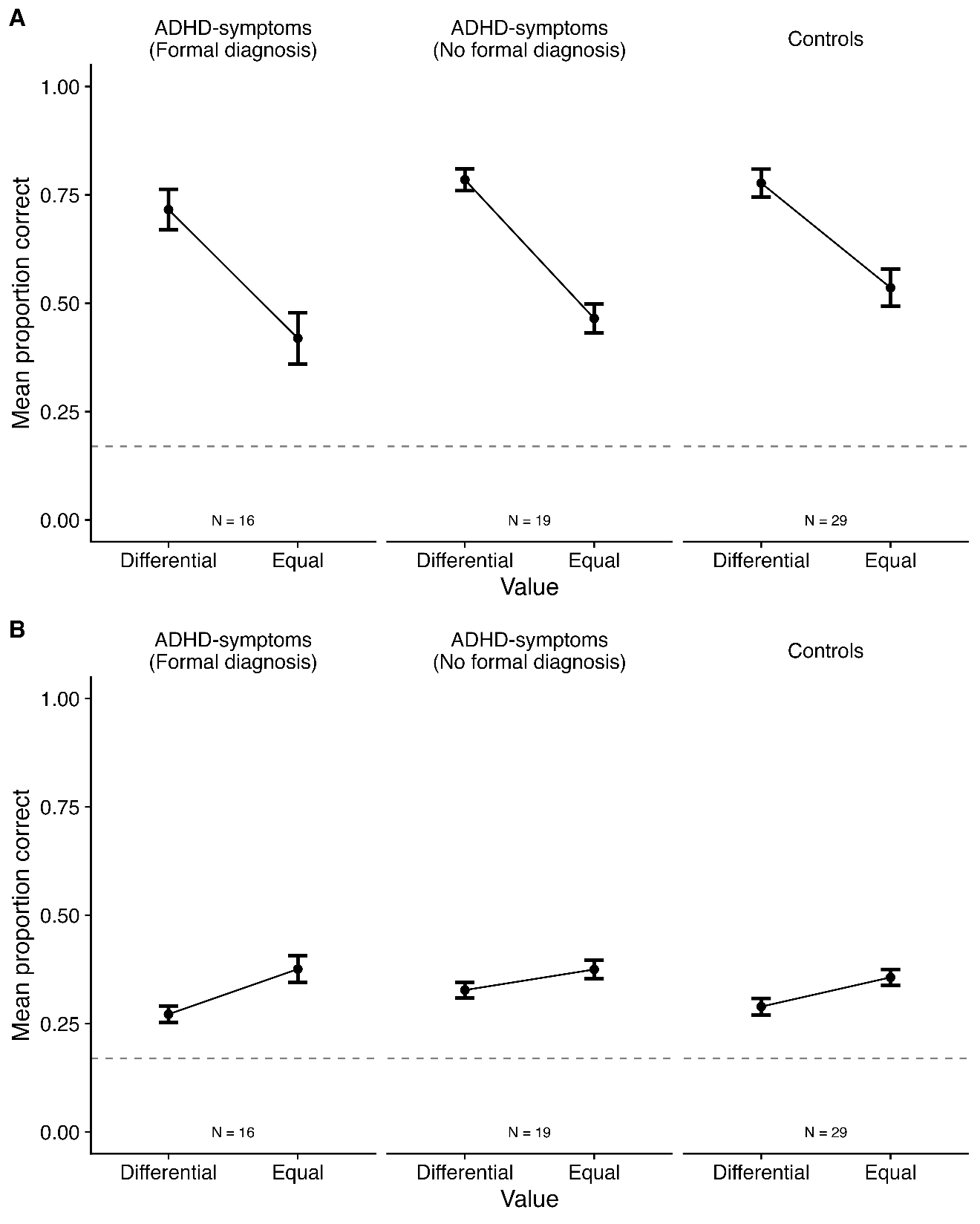


*Figure S.4.1.* Mean proportion correct as a function of value and group in Experiment 1. Panel A displays outcomes at SP1, whilst Panel B displays outcomes at the less valuable SPs. Error bars show standard error. The dotted line at 0.17 indicates chance guessing rate.

1. **Excluding participants receiving medication for ADHD (Experiment 2)**

Five participants in the ADHD-symptoms group reported taking medication for ADHD. Subsidiary analysis was therefore conducted to investigate whether the pattern of results presented in the Results section differed when participants receiving medication for ADHD were excluded. This analysis was conducted on data from 63 participants (29 in the ADHD-symptoms group and 34 in the Control group). As in the main analysis, the groups did not significantly differ on gender (*p* = .160) or years in education (*p* = .151), but the ADHD-symptoms group were significantly younger than the controls (*p* = .002). In the ADHD-symptoms group, six had a formal diagnosis, 22 had no formal diagnosis, and one preferred not to say.

Figure S.5.1.A shows the mean proportion correct at SP1 as a function of value and group in Experiment 2 after excluding participants in the ADHD-symptoms group who reported taking medication for ADHD. Figure S.5.1.B then shows the mean proportion correct at the less valuable SPs as a function of value and group.

**
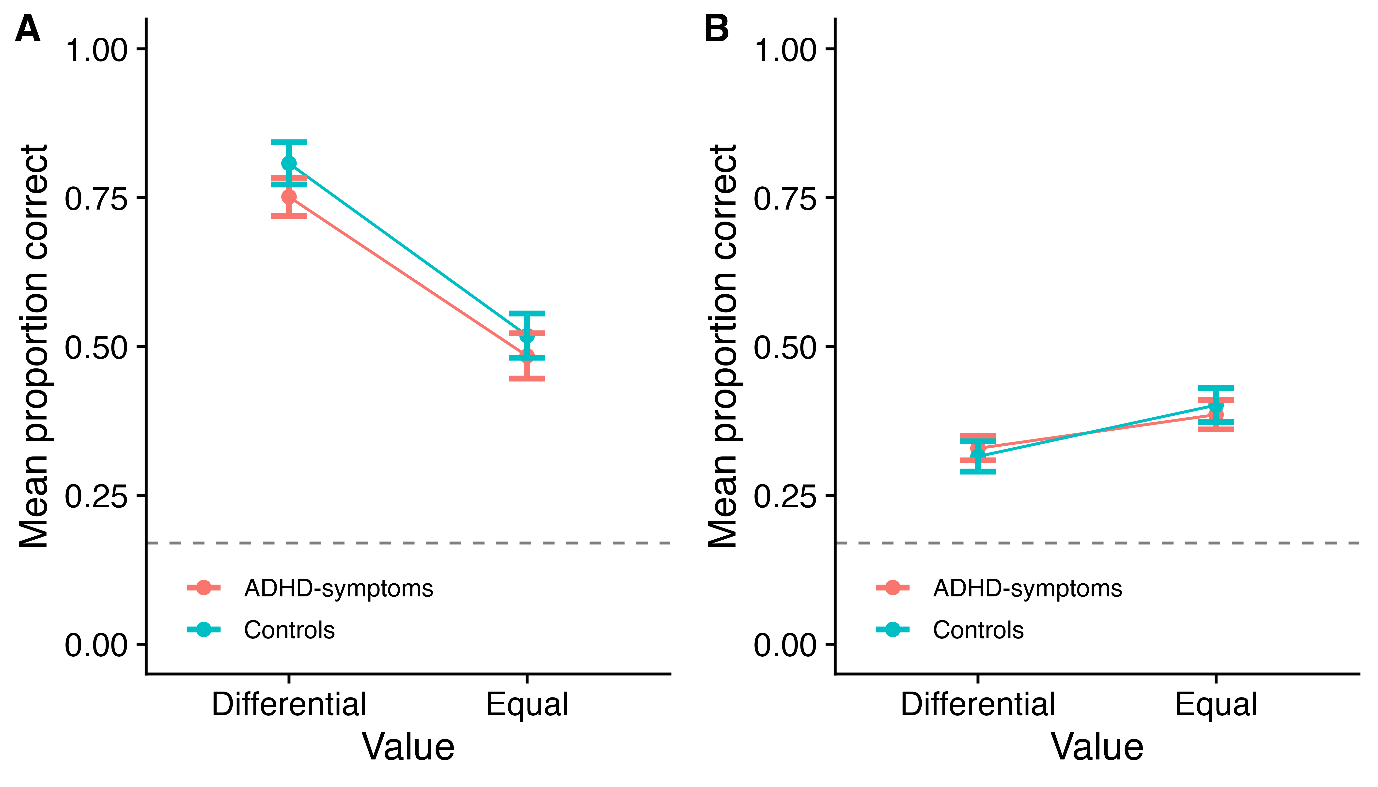
**

*Figure S.5.1.* Mean proportion correct as a function of value and group in Experiment 2. Panel A displays the data at SP1, whilst Panel B displays the data at the less valuable SPs. Error bars show standard error. The dotted line at 0.17 indicates chance guessing rate.

*Performance at SP1*

At SP1, there was a main effect of value (*p* < .001), with higher accuracy in the differential value condition (*M* = 0.78, *SE* = 0.02) relative to the equal value condition (*M* = 0.50, *SE* = 0.03). There was no effect of group (*p* = .234), and no interaction between value and group (*p* = .751).

*Performance at the less valuable SPs*

There was a main effect of value (*p* < .001), with higher accuracy in the equal value condition (*M* = 0.39, *SE* = 0.02) relative to the differential value condition (*M* = 0.32, *SE* = 0.02). There was no effect of group (*p* = .971), and no interaction between value and group (*p* = .333).

Taken together, this demonstrates that the main conclusions of Experiment 2 do not differ when excluding participants who were taking medication for ADHD.

1. **Investigating effects by ADHD diagnosis (Experiment 2)**

Descriptive statistics were computed to investigate whether the pattern of results differed depending on whether participants in the ADHD group had a formal diagnosis of ADHD or not. This plot included all participants included in the main body of the manuscript, except for one participant in the ADHD-symptoms group who was excluded as they responded “rather not to say” to the question asking whether they had a formal diagnosis of ADHD or not. Mean proportion correct is presented in Figure S.6.1., as a function of value and group (Control group, ADHD-symptoms (Formal diagnosis), ADHD-symptoms (No formal diagnosis)). Inferential statistics were not performed due to the small sample sizes for the ADHD-symptoms subgroups (*N* = 11 for the formal diagnosis subgroup and N = 22 for the no formal diagnosis subgroup). As in Experiment 1, it is clear that the broad pattern is similar across groups. Inferential analysis was conducted on the data across both experiments (see supplementary materials section 8).

**
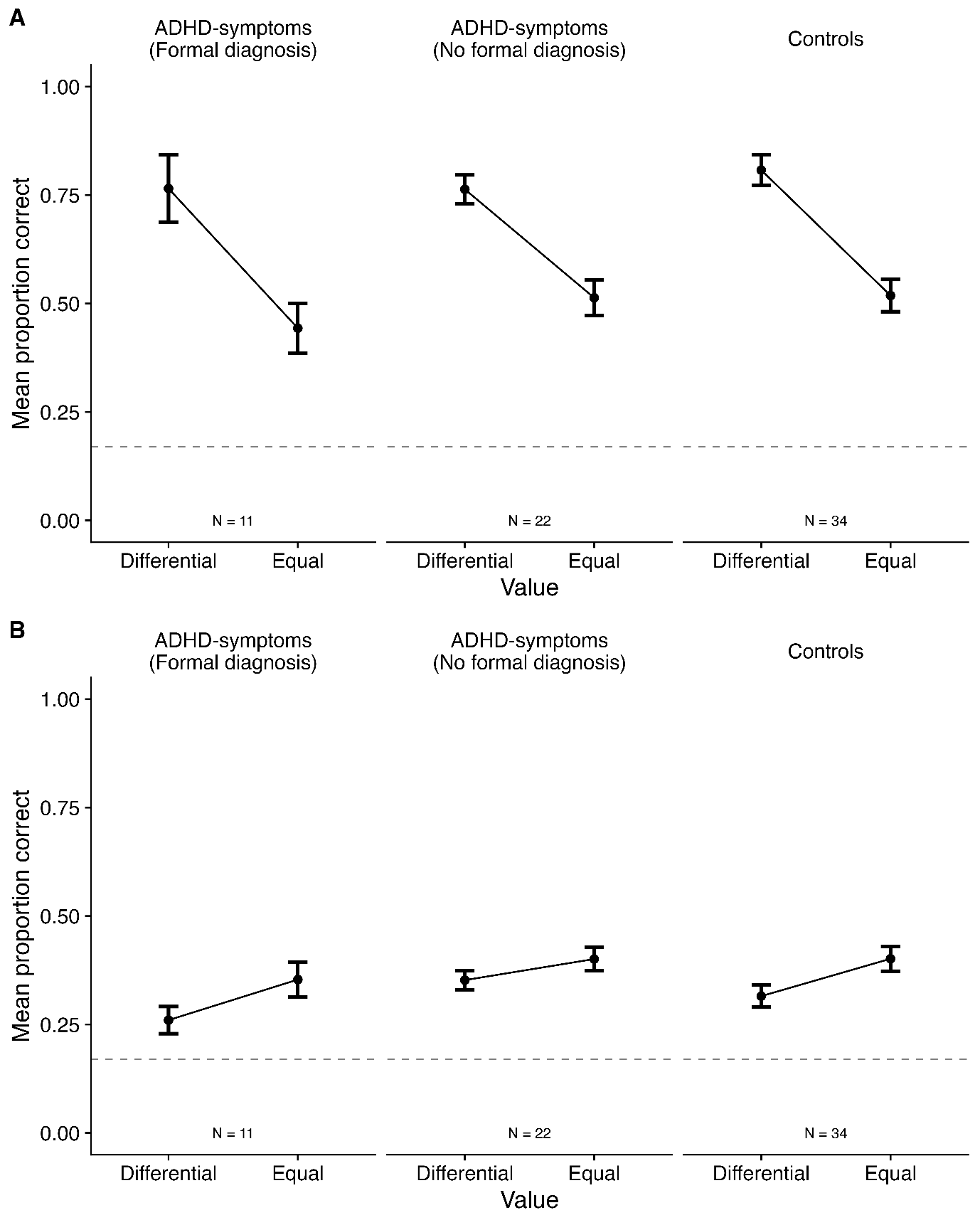
**

*Figure S.6.1.* Mean proportion correct as a function of value, SP, and group in Experiment 2. Panel A displays outcomes at SP1, whereas Panel B displays outcomes at the less valuable SPs. Error bars show standard error. The dotted line at 0.17 indicates chance guessing rate.

1. **Correlations between the ASRS Screener and the prioritization boosts and costs (across experiments)**

As in the analyses of data from the individual experiments, analysis was conducted to investigate whether the degree of inattentive and hyperactive symptoms correlates with the prioritization boost (at SP1) and the cost (at the other SPs). Figure S.7.1., displays scatterplots of ASRS Screener scores, boosts to the high value item, and costs to the low value items.


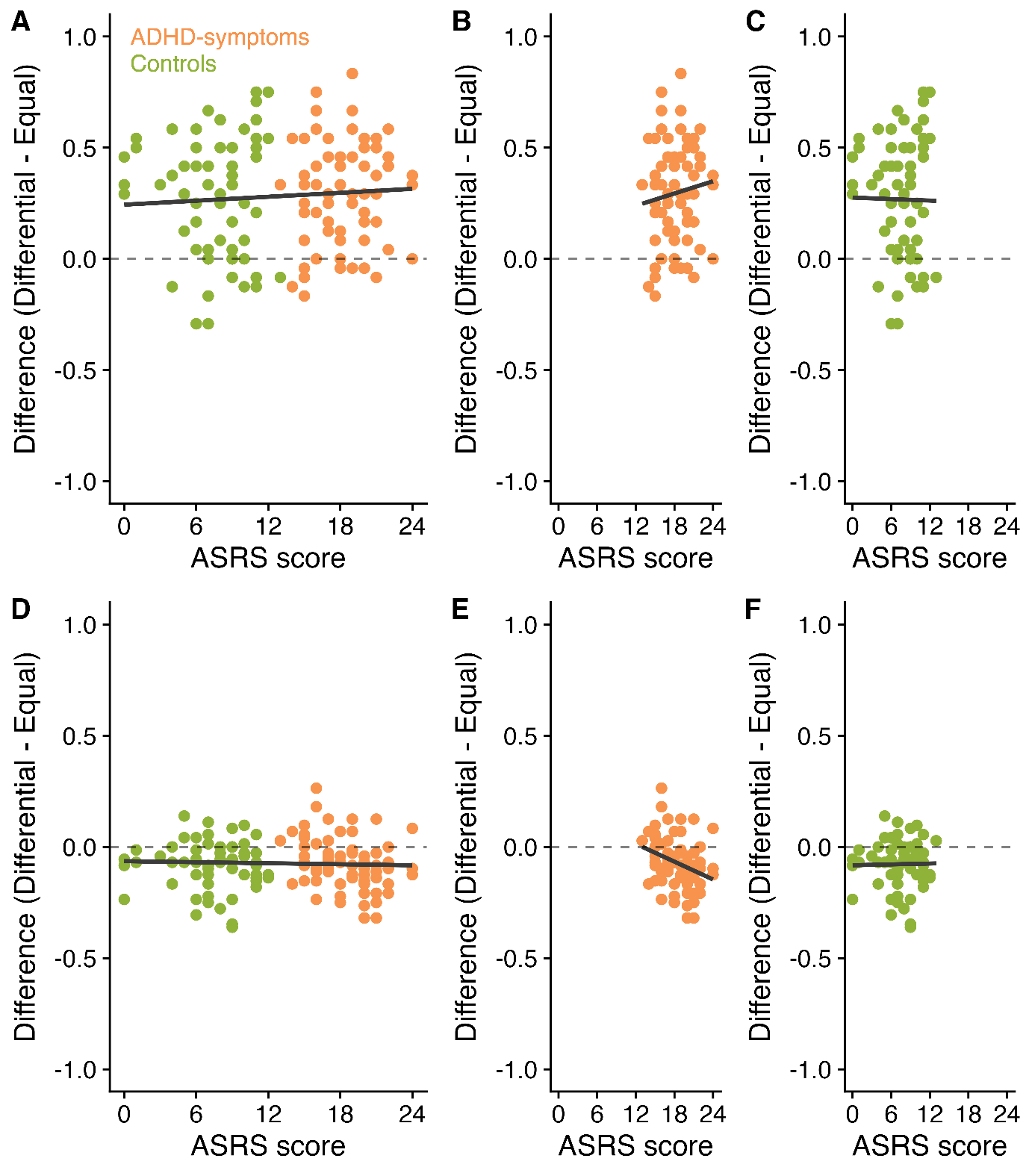


*Figure S.7.1.* Correlations between ASRS Screener scores (reflecting the degree of inattentive and hyperactive behaviours), boosts to SP1, and costs to the less valuable items across experiments. Panel A displays the correlation between ASRS Screener score and the difference in performance between the differential and equal value items at SP1 across participants. Panels B and C then present these correlations for the ADHD-symptoms and Control groups, respectively. Panel D displays the correlation between ASRS Screener score and the difference in performance between the differential and equal value items at the less valuable serial positions. Panels E and F then present these correlations for the ADHD-symptoms and Control groups, respectively.

Pearson's correlation coefficients were conducted to investigate correlations between scores on the ASRS Screener and the prioritization boosts at SP1 and the costs at the less valuable SPs. P-values were corrected using Bonferroni-Holm. The outcomes are presented in Table S.7.1.

*Table S.7.1.* Correlations between the ASRS Screener score, the prioritization boost at SP1 and the prioritization costs at the less valuable SPs (2-4) across experiments.

| Correlation | *r* | *P* | *BF_10_* | *BF_01_* |
| --- | --- | --- | --- | --- |
| ASRS score and prioritization boost at SP1 (all participants) | 0.07 | 1.000 | 0.28 | 3.60 |
| ASRS score and prioritization cost at less valuable SPs (all participants) | -0.04 | 1.000 | 0.22 | 4.46 |
| ASRS score and prioritization boost at SP1 (ADHD-symptoms group) | 0.10 | 1.000 | 0.38 | 2.60 |
| ASRS score and prioritization cost at less valuable SPs (ADHD -symptoms group) | -0.31 | .061 | 5.84 | 0.17 |
| ASRS score and prioritization boost at SP1 (Control group) | -0.01 | 1.000 | 0.29 | 3.48 |
| ASRS score and prioritization cost at less valuable SPs (Control group) | 0.02 | 1.000 | 0.29 | 3.46 |

*Note: p-values corrected using Bonferroni-Holm*

1. **Investigating effects by ADHD diagnosis (across experiments)**

This analysis investigated the effects by ADHD diagnosis across all participants (ignoring experiment). As in the earlier analyses investigating effects by ADHD diagnosis (see supplementary materials sections 4 and 6), three groups were created: Control group, ADHD-symptoms (Formal diagnosis), and ADHD-symptoms (No formal diagnosis). Figure S.8.1.A shows the mean proportion correct at SP1 a function of value and group, Figure S.8.1.B displays mean proportion correct at the less valuable SPs as a function of value and group. This shows a similar pattern across all three groups.


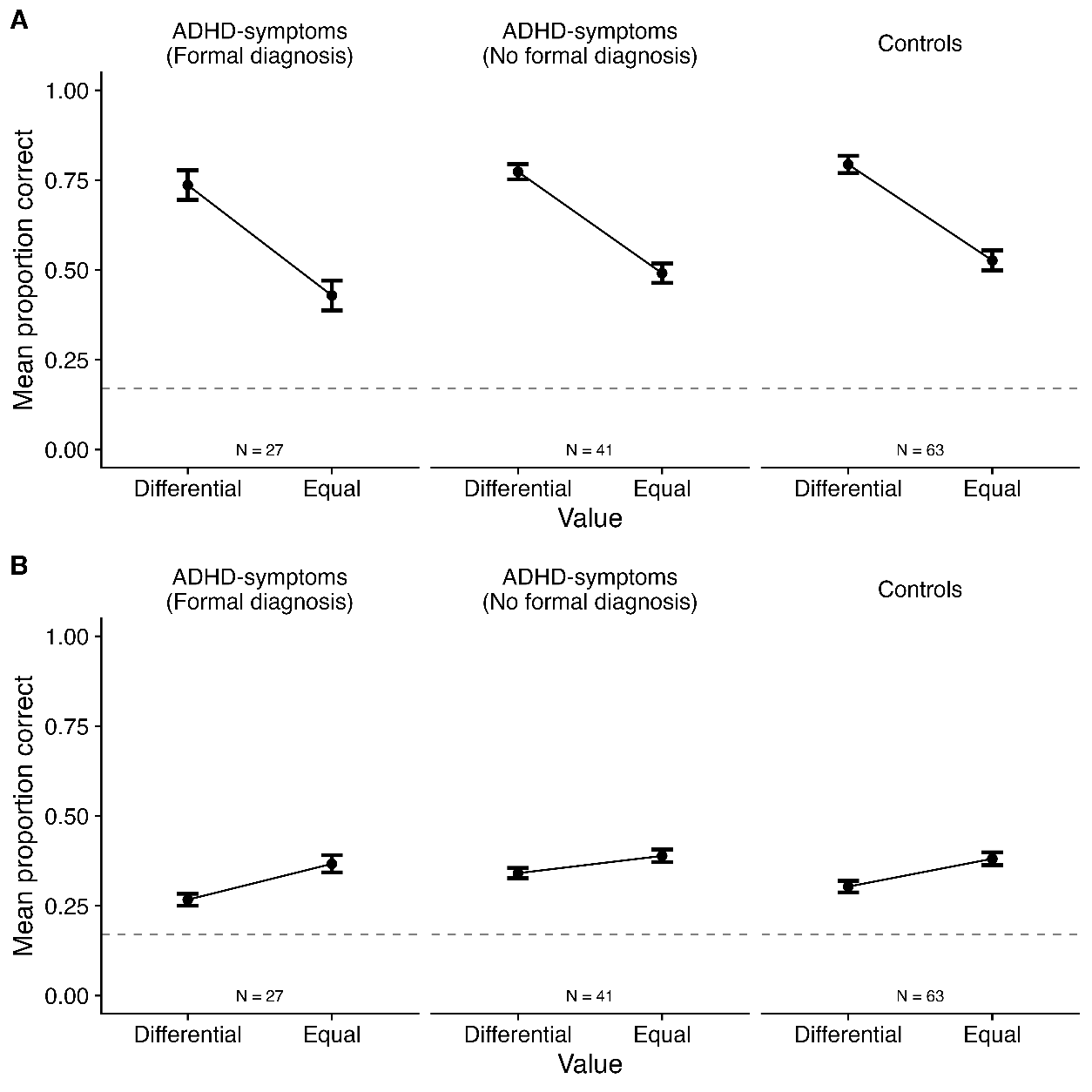


*Figure S.8.1.* Mean proportion correct as a function of value, serial position and group across experiments. Panel A displays the data at SP1, whilst Panel B displays the data at the less valuable SPs. Error bars show standard error. The dotted line at 0.17 indicates chance guessing rate.

*Performance at SP1*

A 2 (Value: differential vs equal; within-subject) x 3 (Group: Control vs ADHD-symptoms (Formal diagnosis) vs ADHD-symptoms (No formal diagnosis; between-subject)) mixed ANOVA was conducted on data at SP1, ignoring the effect of experiment. There was no significant effect of group (*F*(2, 128) = 2.61, *MSE* = 0.04, *p* = .078, $\eta_{p}^{2}$ = .04; *BF_10_* = 0.50, *BF_01_* = 2.00) and no interaction between value and group (*F*(2, 128) = 0.24, *MSE* = 0.03, *p* = .787, $\eta_{p}^{2}$ < .01; *BF_10_* = 0.09, *BF_01_* = 10.64). This analysis did, however, reveal a significant effect of value (*F*(1, 128) = 150.24, *MSE* = 0.03, *p* < .001, $\eta_{p}^{2}$ = .54; *BF_10_* > 10,000), with higher accuracy in the differential value condition (*M* = 0.77, *SE* = 0.02) relative to the equal value condition (*M* = 0.48, *SE* = 0.02).

*Performance at the less valuable SPs*

A 2 (Value: differential vs equal; within-subject) x 3 (Group: Control vs ADHD-symptoms (Formal diagnosis) vs ADHD-symptoms (No formal diagnosis; between-subject)) mixed ANOVA was conducted on data at the less valuable SPs, ignoring the effect of experiment. No significant effect of group emerged (*F*(2, 128) = 1.66, *MSE* = 0.02, *p* = .193, $\eta_{p}^{2}$ = .03; *BF_10_* = 0.41, *BF_01_* = 2.43) and no interaction between value and group (*F*(2, 128) = 1.91, *MSE* = 0.01, *p* = .153, $\eta_{p}^{2}$ = .03; *BF_10_* = 0.35, *BF_01_* = 2.85). This analysis did, however, reveal a significant effect of value (*F*(1, 128) = 54.35, *MSE* = 0.01, *p* < .001, $\eta_{p}^{2}$ = .30; *BF_10_* > 10,000), with higher accuracy in the equal value condition (*M* = 0.38, *SE* = 0.01) to the differential value condition (*M* = 0.30, *SE* = 0.01).

1. **Three-way ANOVAs**

Three-way ANOVAs were also performed on the dataset to investigate whether significant interactions emerged between value, SP, and group. This allows a more detailed investigation of the costs to less valuable SPs, which may not be equivalent across SPs. We note, however, that caution should be taken when interpreting these findings, as our power calculation was based on the simpler 2 (Value) x 2 (Group) analytical approach taken in the main body of the manuscript.

**Experiment 1**

Mean proportion correct as a function of value, SP, and group is presented in Figure S.9.1. A 2 (Value: differential vs equal; within-subject) x 4 (SP: 1-4; within-subject) x 2 (Group: ADHD-symptoms vs control; between-subjects) mixed ANOVA revealed no main effect of value (*F*(1, 63) = 1.82, *MSE* = 0.02, *p* = .182, $\eta_{p}^{2}$ = .03; *BF_10_*  = 0.20, *BF_01_*  = 5.07) or group (*F*(1, 63) = 0.01, *MSE* = 0.06, *p* = .927, $\eta_{p}^{2}$ < .01; *BF_10_*  = 0.16, *BF_01_*  = 6.44). There was, however, a significant effect of SP (*GG-corrected* *F*(2.37, 149.20) = 119.93, *MSE* = 0.03, *p* < .001, $\eta_{p}^{2}$ = .66; *BF_10_* > 10,000). Bonferroni-Holm corrected t-tests revealed that performance at SP1 (*M* = .63, *SE* = .02) was superior to performance at SP2 (*M* = .35, *SE* = .02; *p* < .001; *BF_10_*  > 10,000), SP3 (*M* = .26, *SE* = .01; *p* < .001; *BF_10_*  > 10,000), and SP4 (*M* = .39, *SE* = .02; *p* < .001; *BF_10_*  > 10,000). Moreover, performance at SP3 was significantly worse than performance at SP2 (*p* < .001; *BF_10_* = 1248.52) and SP4 (*p* < .001; *BF_10_*  > 10,000). There was no significant difference between performance at SP2 and SP4 (*p* = .139; *BF_10_*  = 0.48, *BF_10_*  = 2.09). There was a significant two-way interaction between value and SP (*GG-corrected* *F*(2.22, 140.03) = 50.75, *MSE* = 0.03, *p* < .001, $\eta_{p}^{2}$ = .45, *BF_10_* > 10,000). There was also a significant interaction between group and SP (*GG-corrected* *F*(2.37, 149.20) = 3.39, *MSE* = 0.03, *p* = .029, $\eta_{p}^{2}$ = .05, *BF_10_* = 2.90). There was no two-way interaction between value and group (*F*(1, 63) = 0.27, *MSE* = 0.02, *p* = .603, $\eta_{p}^{2}$ < .01, *BF_10_* = 0.15, *BF_01_* = 6.57), and crucially no three-way interaction between value, SP and group (*GG-corrected* *F*(2.22, 140.03) = 1.47, *MSE* = 0.03, *p* = .232, $\eta_{p}^{2}$ = .02; *BF_10_* = 0.21, *BF_01_* = 4.78). The BF analysis indicated that the best model included a main effect of SP, an interaction between value and SP, and an interaction between SP and group (*BF_10_* > 10,000 relative to the null model containing participant only).

**
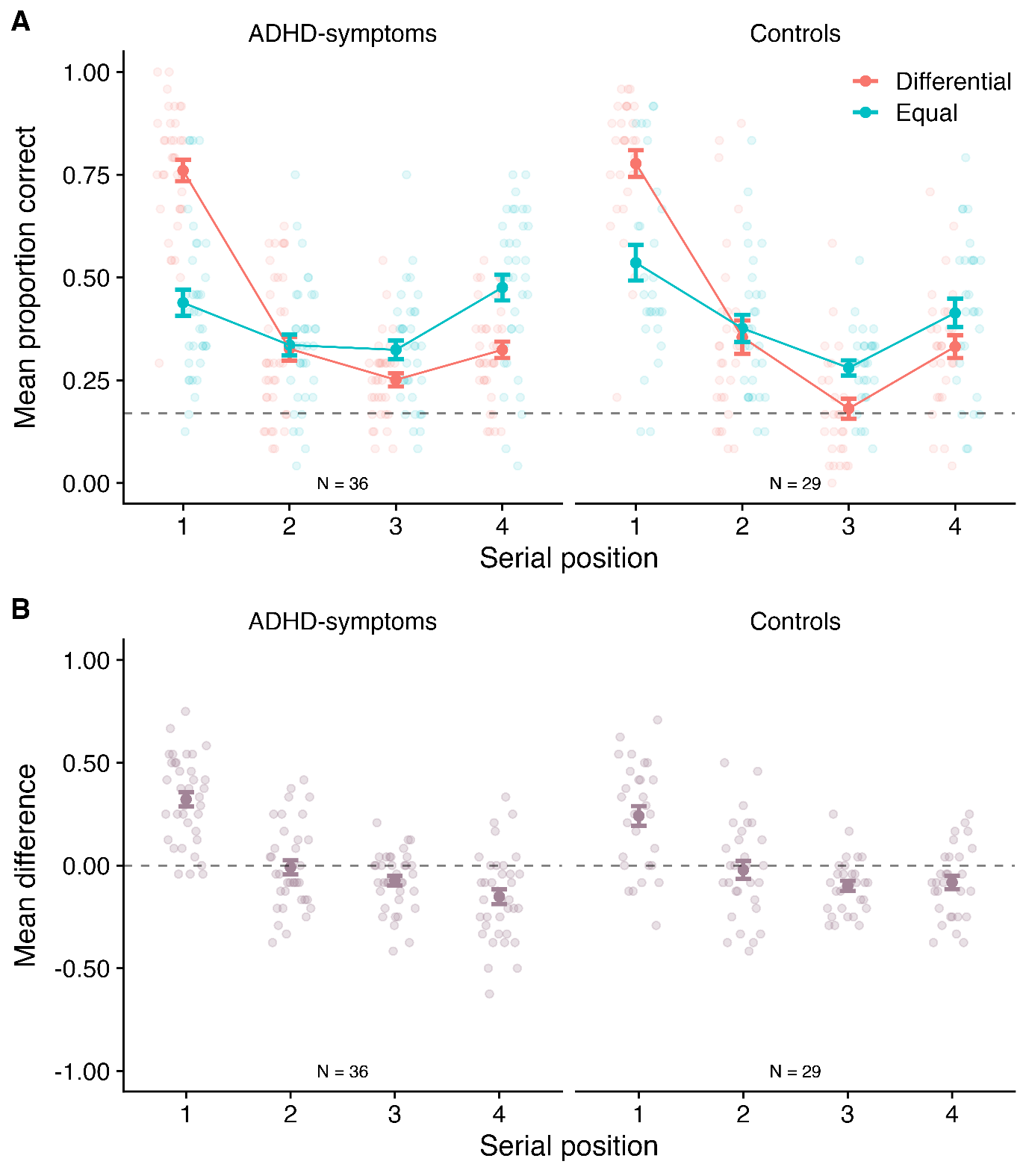
**

*Figure S.9.1.* Outcomes in Experiment 1. Panel A: Mean proportion correct as a function of value, SP and group. The bold point denotes mean across participants, whilst error bars denote standard error. The lighter points in the background reflect the average performance of individual participants. The dotted line at 0.17 reflects chance guessing rate, based on a stimulus set of six items. Panel B: The mean difference between the differential and equal value conditions, as a function of SP and group (calculating as Differential – Equal at each SP). The bold point reflects mean difference across participants, whilst error bars display standard error. The lighter points in the background reflect the mean difference for individual participants. The dotted line at 0.00 reflects no difference.

To understand the interaction between value and SP, paired sample t-tests were conducted on data at each SP (corrected using Bonferroni-Holm). At SP1, performance was better in the differential value condition (*M* = .77, *SE* = .02) relative to the equal value condition (*M* = .49, *SE* = .03; *p* < .001, *d* = 1.22; *BF_10_* > 10,000). The opposite pattern emerged at SP3 (differential *M* = .22, *SE* = .01; equal *M* = .30, *SE* = .02; *p* < .001, *d* = -0.61; *BF_10_* = 3039.62) and SP4 (differential *M* = .33, *SE* = .02; equal *M* = .45, *SE* = .02; *p* < .001, *d* = -0.61; *BF_10_* = 2394.19). There was no significant difference between value conditions at SP2 (*p* = .579, *d* = -0.07; *BF_10_* = 0.16; *BF_01_* = 6.39).

To understand the interaction between group and SP, t-tests were conducted to investigate whether there was a significant difference between groups at each SP. No significant differences emerged following Bonferroni-Holm correction, although the difference at SP3 did approach significance (SP3 difference *p* = .080, *d* = 0.46, *BF_10_* = 3.78; at other SPs *p* ≥ .380, *d* ≥ -0.24 and ≤ 0.16, *BF_10_* ≤ 0.44, *BF_01_* ≥ 2.28).

**Experiment 2**

Mean accuracy as a function of value, SP, and group is displayed in Figure S.9.2.A. The mean difference between value conditions as a function of SP and group is displayed in Figure S.9.2.B. A 2 (Value: differential vs equal; within-subject) x 4 (SP: 1-4; within-subject) x 2 (Group: ADHD-symptoms vs control; between-subjects) mixed ANOVA revealed a significant effect of SP (*GG-corrected* *F*(2.43, 160.13) = 111.23, *MSE* = 0.03, *p* < .001, $\eta_{p}^{2}$ = .63; *BF_10_* > 10,000). Bonferroni-Holm corrected post-hoc comparisons revealed higher accuracy at SP1 (*M* = .64, *SE* = .02) relative to SP2 (*M* = .35, *SE* = .02, *p* < .001; *BF_10_* > 10,000), SP3 (*M* = .31, *SE* = .02, *p* < .001; *BF_10_* > 10,000), and SP4 (M = .40, *SE* = .02, *p* < .001; *BF_10_* > 10,000). Moreover, performance at SP4 was better than SP2 (*p* = .038; *BF_10_* = 1.10) and SP3 (*p* < .001; *BF_10_* > 10,000). Finally, performance at SP2 was superior to performance at SP3 (*p* = .022; *BF_10_* = 3.18). There was no effect of value (*F*(1, 66) = 1.03, *MSE* = 0.02, *p* = .313, $\eta_{p}^{2}$ = .02; *BF_10_* = 0.16, *BF_01_* = 6.17) or group (*F*(1, 66) = 0.38, *MSE* = 0.11, *p* = .539, $\eta_{p}^{2}$ = .01; *BF_10_* = 0.23; *BF_01_* = 4.32). There was no interaction between value and group *(F*(1, 66) = 0.20, *MSE* = 0.02, *p* = .658 $\eta_{p}^{2}$ < .01; *BF_10_* = 0.15; *BF_01_* = 6.78). There was also no interaction between group and SP in this experiment (*GG-corrected* *F*(2.43, 160.13) = 0.55, *MSE* = 0.03, *p* = .611, $\eta_{p}^{2}$ = .01; *BF_10_* = 0.04; *BF_01_* = 25.84). There was, however, a significant interaction between value and SP (*GG-corrected* *F*(1.96, 129.25) = 56.92, *MSE* = 0.03, *p* < .001, $\eta_{p}^{2}$ = .46; *BF_10_* > 10,000). Finally, there was no three-way interaction between value, SP and group (*GG-corrected* *F*(1.96, 129.25) = 0.18, *MSE* = 0.03, *p* = .833, $\eta_{p}^{2}$ < .01; *BF_10_* = 0.05, *BF_01_* = 21.45). The Bayes Factor analysis indicated that the best model included a main effect of SP, as well as interaction between value and SP (*BF_10_* > 10,000 relative to the null model containing participant only).

To investigate the interaction between value and SP, four Bonferroni-Holm corrected paired sample t-tests were conducted to examine the effect of value at each SP (averaged over group). At SP1, performance was significantly higher in the differential value condition (*M* = .78, *SE* = .02) relative to the equal value condition (*M* = .50, *SE* = .02; *p* < .001; *d* = 1.05; *BF_10_* > 10,000). The opposite pattern was observed at SP3 (differential value *M* = .26, *SE* = .02; equal value *M* = .36, *SE* = .02; *p* < .001; *d* = -0.63, *BF_10_* = 7384.84) and SP4 (differential value *M* = .35, *SE* = .02; equal value *M* = .45, *SE* = .02; *p* = .001; *d* = -0.47, *BF_10_* = 87.75). No significant difference was observed at SP2 (*p* = .099; *d* = -0.20, *BF_10_* = 0.51; *BF_01_* = 1.97).

**
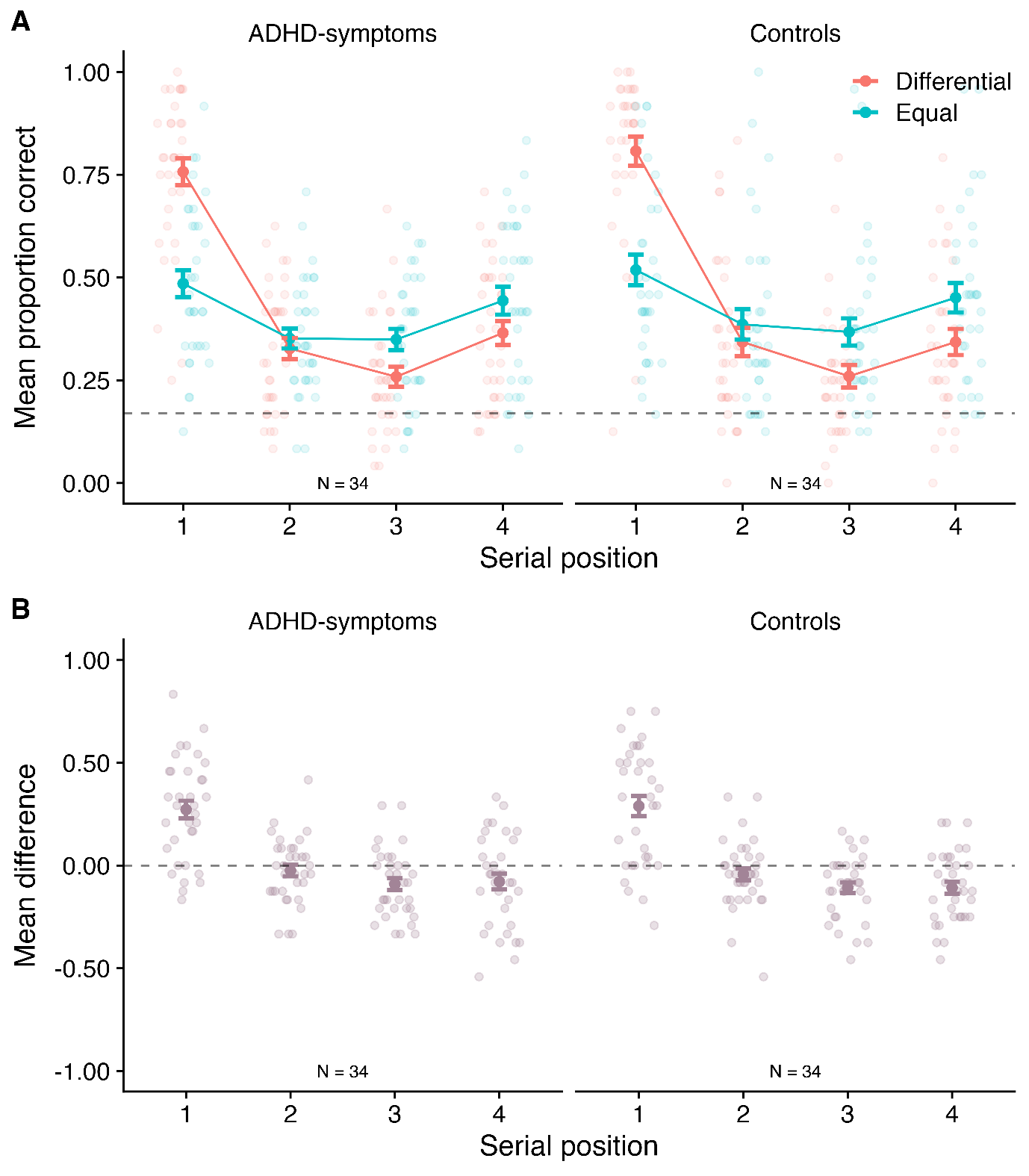
**

*Figure S.9.2.* Outcomes in Experiment 2. Panel A: Mean proportion correct as a function of value, SP and group. The bold point denotes mean across participants, whilst error bars denote standard error. The lighter points in the background reflect the average performance of individual participants. The dotted line at 0.17 reflects chance guessing rate, based on a stimulus set of six items. Panel B: The mean difference between the differential and equal value conditions, as a function of SP and group (calculating as Differential – Equal at each SP). The bold point reflects mean difference across participants, whilst error bars display standard error. The lighter points in the background reflect the mean difference for individual participants. The dotted line at 0.00 reflects no difference.

**Across experiments**

Figure S.9.3 shows the mean proportion correct across experiments as a function of value, SP, and group. A 2 (Value: differential vs equal; within-subject) x 2 (Group: ADHD-symptoms vs control; between-subject) x 4 (SP: 1-4; within-subject) mixed design was conducted, ignoring experiment. The ANOVA revealed no main effect of group (*F*(1, 131) = 0.39, *MSE* = 0.08, *p* = .535, $\eta_{p}^{2}$ = < .01; *BF_10_* = 0.15, *BF_01_* = 6.56) or value (*F*(1, 131) = 2.75, *MSE* = 0.02, *p* = .099, $\eta_{p}^{2}$ = .02; *BF_10_* = 0.24, *BF_01_* = 4.23). There was, however, a main effect of SP (*GG-corrected* *F*(2.45, 320.35) = 230.09, *MSE* = 0.03, *p* < .001, $\eta_{p}^{2}$ = .64; *BF_10_* > 10,000). This was driven by significant differences between all SPs (*p* < .001 and *BF_10_* > 10,000 for all comparisons following Bonferroni-Holm correction, except the difference between SP2 and SP4, where *p* = .011 and *BF_10_* = 2.82). There was a significant interaction between value and SP (*GG-corrected* *F*(2.14, 280.00) = 108.94, *MSE* = 0.03, *p* < .001, $\eta_{p}^{2}$ = .45; *BF_10_* > 10,000). There was also a marginally significant interaction between group and SP (*GG-corrected* *F*(2.45, 320.35) = 2.95, *MSE* = 0.03, *p* = .043, $\eta_{p}^{2}$ = .02), although this was not supported by the BF analysis (*BF_10_* = 0.77, *BF_01_* = 1.30). There was no interaction between value and group (*F*(1, 131) = 0.48, *MSE* = 0.02, *p* = .491, $\eta_{p}^{2}$ < .01; *BF_10_* = 0.12, *BF_01_* = 8.36), and no three-way interaction between value, group, and SP (*GG-corrected* *F*(2.14, 280.00) = 0.41, *MSE* = 0.03, *p* = .677, $\eta_{p}^{2}$ < .01; *BF_10_* = 0.03, *BF_01_* = 35.35).


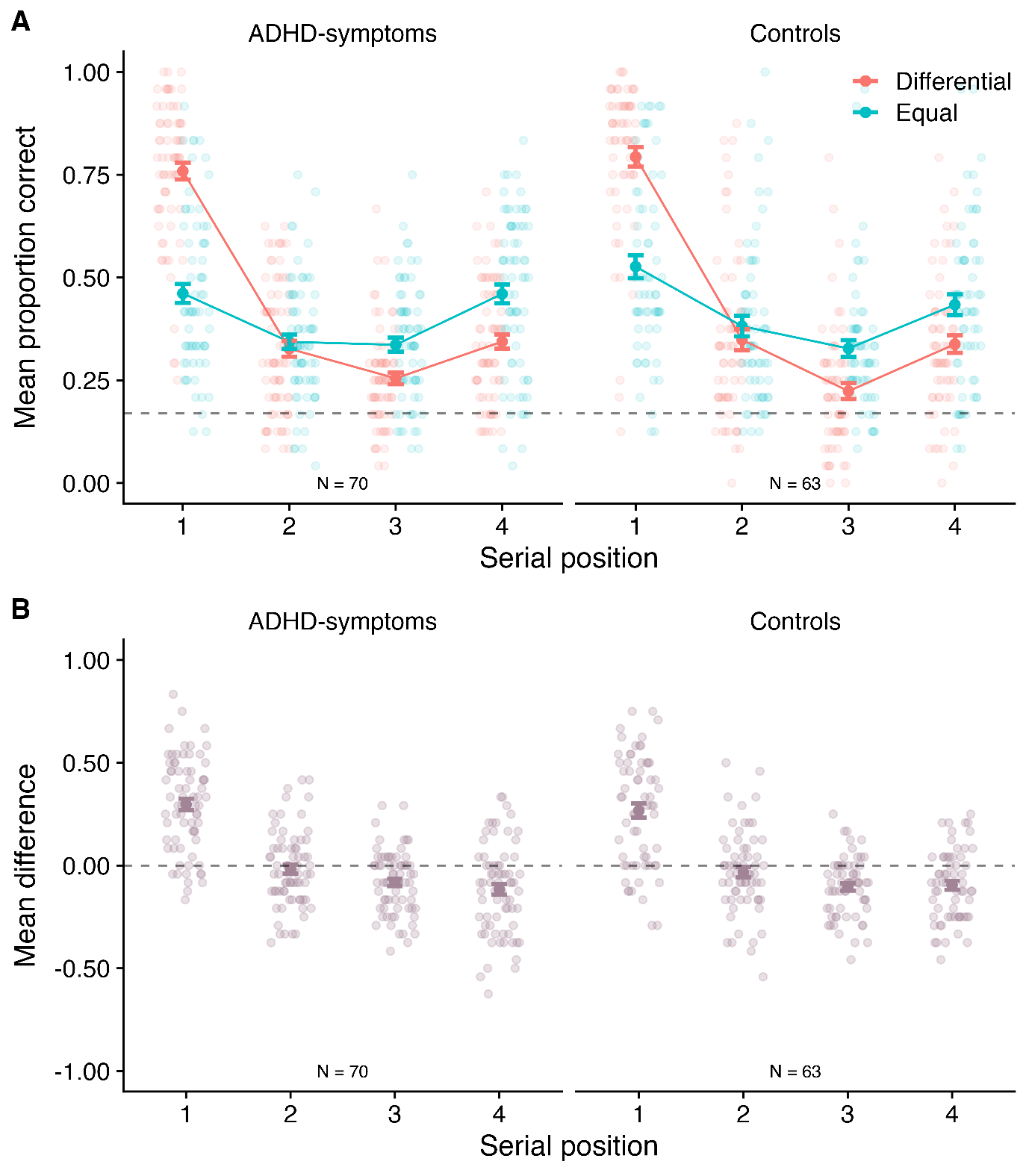


*Figure S.9.3.* Outcomes when the data for both experiments are combined. Panel A: Mean proportion correct as a function of value, SP and group. The bold point denotes mean across participants, whilst error bars denote standard error. The lighter points in the background reflect the average performance of individual participants. The dotted line at 0.17 reflects chance guessing rate, based on a stimulus set of six items. Panel B: The mean difference between the differential and equal value conditions, as a function of SP and group (calculating as Differential – Equal at each SP). The bold point reflects mean difference across participants, whilst error bars display standard error. The lighter points in the background reflect the mean difference for individual participants. The dotted line at 0.00 reflects no difference.

To investigate the key interaction between value and SP, Bonferroni-Holm corrected paired sample t-tests were conducted to examine the effect of value at each SP (averaged over group). At SP1, performance was significantly higher in the differential value condition (*M* = .78, *SE* = .02) relative to the equal value condition (*M* = .49, *SE* = .02; *p* < .001; *d* = 1.13; *BF_10_* > 10,000). Meanwhile, the reverse was found at SP3 (differential value *M* = .24, *SE* = .01; equal value *M* = .33, *SE* = .01; *p* < .001; *d* = -0.62, *BF_10_* > 10,000) and SP4 (differential value *M* = .34, *SE* = .01; equal value *M* = .45, *SE* = .02; *p* < .001; *d* = -0.54, *BF_10_* > 10,000). No significant difference was observed at SP2 (*p* = .142; *d* = -0.13, *BF_10_* = 0.27; *BF_01_* = 3.69).

To understand the interaction between group and SP, t-tests were conducted to investigate whether there was a significant difference between groups at each SP. No significant differences emerged following Bonferroni-Holm correction (*p* ≥ .217, *d* ≥ -0.21 and ≤ 0.13, *BF_10_* ≤ 0.52, *BF_01_* ≥ 1.91).

1. **Investigating the effects of trial-by-trial feedback by comparing the experiments**

Figure S.10.1.A shows the mean proportion correct at SP1 a function of value and experiment, meanwhile Figure S.10.1.B displays the mean proportion correct at the less valuable SPs a function of value and experiment.


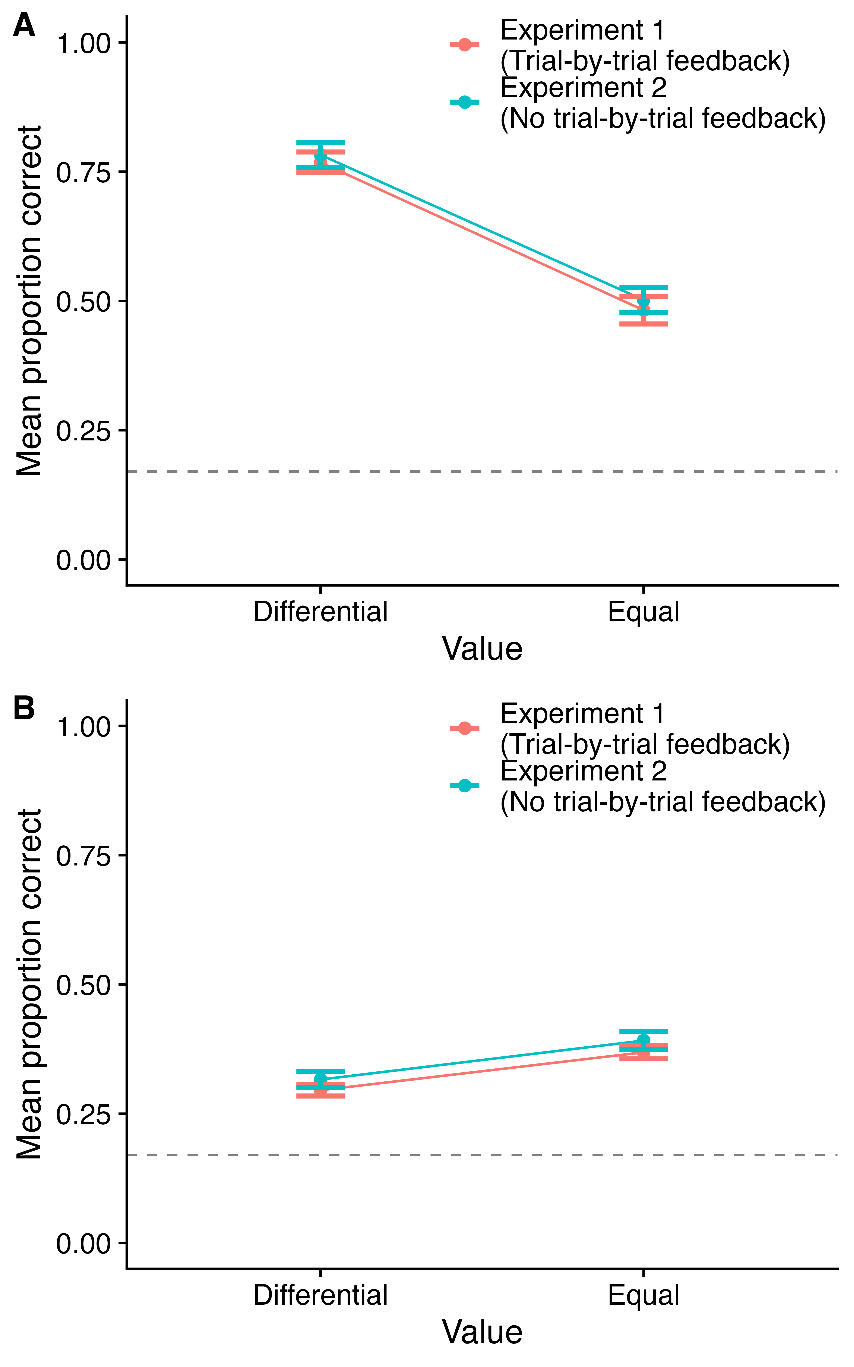


*Figure S.10.1* Mean proportion correct as a function of value, SP, and experiment (ignoring group). Panel A shows data for SP1, whereas Panel B shows data for the less valuable SPs. Error bars show standard error. The dotted line at 0.17 indicates chance guessing rate.

*Performance at SP1*

A 2 (Value: differential vs equal; within-subject) x 2 (Experiment: Experiment 1 [trial-by-trial feedback] vs Experiment 2 [no trial-by-trial feedback; between-subjects] mixed ANOVA was conducted. The effect of group was ignored in this analysis, given the absence of a group effect or interaction with group in any of the previous analyses in the main body and supplementary materials. There was a significant effect of value *F*(1, 131) = 167.43, *MSE* = 0.03, *p* < .001, $\eta_{p}^{2}$ = .56; *BF_10_* > 10,000) with higher accuracy in the differential value condition (*M* = 0.78, *SE* = 0.02) relative to the equal value condition (*M* = 0.49, *SE* = 0.02). There was no significant effect of experiment (*F*(1, 131) = 0.44, *MSE* = 0.04, *p* = .511, $\eta_{p}^{2}$ < .01; *BF_10_* = 0.20, *BF_01_* = 4.99) and no interaction between value and experiment (*F*(1, 131) = 0.01, *MSE* = 0.03, *p* = .905, $\eta_{p}^{2}$ < .01; *BF_10_* = 0.19, *BF_01_* = 5.38).

*Performance at the less valuable SPs*

A 2 (Value: differential vs equal; within-subject) x 2 (Experiment: Experiment 1 [trial-by-trial feedback] vs Experiment 2 [no trial-by-trial feedback; between-subjects] mixed ANOVA was conducted on data for the less valuable SPs. As described above, the “group” variable was ignored in this analysis. A significant effect of value was observed (*F*(1, 131) = 59.31, *MSE* = 0.01, *p* < .001, $\eta_{p}^{2}$ = .31; *BF_10_* > 10,000) with higher accuracy in the equal value condition (*M* = 0.78, *SE* = 0.02) relative to the differential value condition (*M* = 0.49, *SE* = 0.02). There was no significant effect of experiment (*F*(1, 131) = 1.37, *MSE* = 0.02, *p* = .244, $\eta_{p}^{2}$ = .01; *BF_10_* = 0.44, *BF_01_* = 2.26). There was also no significant interaction between value and experiment (*F*(1, 131) = 0.01, *MSE* = 0.01, *p* = .914, $\eta_{p}^{2}$ < .01; *BF_10_* = 0.19, *BF_01_* = 5.35).
